# Child morbidity and mortality associated with alternative policy responses to the economic crisis in Brazil: A nationwide microsimulation study

**DOI:** 10.1371/journal.pmed.1002570

**Published:** 2018-05-22

**Authors:** Davide Rasella, Sanjay Basu, Thomas Hone, Romulo Paes-Sousa, Carlos Octávio Ocké-Reis, Christopher Millett

**Affiliations:** 1 Instituto de Saúde Coletiva, Universidade Federal da Bahia, Salvador, Bahia, Brazil; 2 Public Health Policy Evaluation Unit, Department of Primary Care and Public Health, School of Public Health, Imperial College London, London, United Kingdom; 3 Center for Population Health Sciences, School of Medicine, Stanford University, Stanford, California, United States of America; 4 Center for Primary Care and Outcomes Research, School of Medicine, Stanford University, Stanford, California, United States of America; 5 Department of Health Research and Policy, School of Medicine, Stanford University, Stanford, California, United States of America; 6 Center for Primary Care, Harvard Medical School, Boston, Massachusetts, United States of America; 7 René Rachou Institute, Fiocruz Minas, Belo Horizonte, Minas Gerais, Brasil; 8 Institute for Applied Economic Research, Rio de Janeiro, Brazil; 9 Center for Epidemiological Studies in Health and Nutrition, University of São Paulo, São Paulo, Brazil; London School of Hygiene and Tropical Medicine, UNITED KINGDOM

## Abstract

**Background:**

Since 2015, a major economic crisis in Brazil has led to increasing poverty and the implementation of long-term fiscal austerity measures that will substantially reduce expenditure on social welfare programmes as a percentage of the country’s GDP over the next 20 years. The Bolsa Família Programme (BFP)—one of the largest conditional cash transfer programmes in the world—and the nationwide primary healthcare strategy (Estratégia Saúde da Família [ESF]) are affected by fiscal austerity, despite being among the policy interventions with the strongest estimated impact on child mortality in the country. We investigated how reduced coverage of the BFP and ESF—compared to an alternative scenario where the level of social protection under these programmes is maintained—may affect the under-five mortality rate (U5MR) and socioeconomic inequalities in child health in the country until 2030, the end date of the Sustainable Development Goals.

**Methods and findings:**

We developed and validated a microsimulation model, creating a synthetic cohort of all 5,507 Brazilian municipalities for the period 2017–2030. This model was based on the longitudinal dataset and effect estimates from a previously published study that evaluated the effects of poverty, the BFP, and the ESF on child health. We forecast the economic crisis and the effect of reductions in BFP and ESF coverage due to current fiscal austerity on the U5MR, and compared this scenario with a scenario where these programmes maintain the levels of social protection by increasing or decreasing with the size of Brazil’s vulnerable populations (policy response scenarios). We used fixed effects multivariate regression models including BFP and ESF coverage and accounting for secular trends, demographic and socioeconomic changes, and programme duration effects. With the maintenance of the levels of social protection provided by the BFP and ESF, in the most likely economic crisis scenario the U5MR is expected to be 8.57% (95% CI: 6.88%–10.24%) lower in 2030 than under fiscal austerity—a cumulative 19,732 (95% CI: 10,207–29,285) averted under-five deaths between 2017 and 2030. U5MRs from diarrhoea, malnutrition, and lower respiratory tract infections are projected to be 39.3% (95% CI: 36.9%–41.8%), 35.8% (95% CI: 31.5%–39.9%), and 8.5% (95% CI: 4.1%–12.0%) lower, respectively, in 2030 under the maintenance of BFP and ESF coverage, with 123,549 fewer under-five hospitalisations from all causes over the study period. Reduced coverage of the BFP and ESF will also disproportionately affect U5MR in the most vulnerable areas, with the U5MR in the poorest quintile of municipalities expected to be 11.0% (95% CI: 8.0%–13.8%) lower in 2030 under the maintenance of BFP and ESF levels of social protection than under fiscal austerity, compared to no difference in the richest quintile. Declines in health inequalities over the last decade will also stop under a fiscal austerity scenario: the U5MR concentration index is expected to remain stable over the period 2017–2030, compared to a 13.3% (95% CI: 5.6%–21.8%) reduction under the maintenance of BFP and ESF levels of protection. Limitations of our analysis are the ecological nature of the study, uncertainty around future macroeconomic scenarios, and potential changes in other factors affecting child health. A wide range of sensitivity analyses were conducted to minimise these limitations.

**Conclusions:**

The implementation of fiscal austerity measures in Brazil can be responsible for substantively higher childhood morbidity and mortality than expected under maintenance of social protection—threatening attainment of Sustainable Development Goals for child health and reducing inequality.

## Abstract 2

In a modeling study, Davide Rasella and colleagues investigate the possible consequences for child illness and mortality of different levels of coverage by social protection programs in Brazil.

## Author summary

**Why was this study done?:**

- There is little evidence on the health impacts of economic crisis and fiscal austerity measures in low- and middle-income countries with fragile social protection systems and high poverty rates. Furthermore, there is poor understanding of how austerity measures could threaten attainment of the Sustainable Development Goals (SDGs) in these countries.
- A platform of long-term fiscal austerity measures is underway in Brazil in response to acute economic and political crises. Little is known about the possible impact of the planned reductions (as percentage of country GDP) in expenditure on social welfare programmes over the next two decades on health outcomes.

**What did the researchers do and find?:**

- We forecast the effects of the economic crisis and austerity measures on two key social welfare programmes (the Bolsa Família Programme [BFP] and Estratégia Saúde da Família [ESF]) and on child morbidity and mortality in Brazil using a synthetic cohort of 5,507 municipalities using a robust modelling technique—discrete-time microsimulation—with municipality-specific time trends and parameters.
- Our forecasts indicate that over the period 2017–2030, reducing coverage of the BFP and ESF, compared with maintaining their coverage, could result in a higher child mortality rate—up to 8.6% higher in 2030. These austerity measures would then be responsible for almost 20,000 avoidable childhood deaths and 124,000 avoidable childhood hospitalisations between 2017 and 2030.
- According to our estimates, poorer municipalities would be disproportionately affected, ensuring that Brazil’s already high inequalities would persist until at least 2030. In contrast, maintaining BFP and ESF coverage would contribute to reducing these high inequalities, in line with the SDGs.
- A range of sensitivity analyses show that, even under different simulated intensities and durations of economic crisis and reductions in BFP and ESF coverage, the numbers of avoidable childhood deaths and hospitalisations under austerity measures are expected to be high.

**What do these findings mean?:**

- Our study suggests that reduced coverage of poverty-alleviation and primary care programmes may result in a substantial number of preventable child deaths and hospitalisations in Brazil.
- These austerity measures will disproportionately impact child mortality in the poorest municipalities, disrupting previous declines in inequality in child health outcomes.
- The implementation of austerity measures in LMICs during economic crises is likely to threaten achievement of the SDGs related to poverty reduction, improving health, and reducing health inequality.

## Introduction

Several studies have examined the effects of economic crises on health outcomes in high-income countries [1], but very little evidence covers low- and middle-income countries (LMICs). Some studies suggest that economic crises in LMICs may have particularly detrimental impacts on child health [2,3], but evidence remains sparse. This is an important knowledge gap given that the global economic crisis has now affected many LMICs and may impede progress towards the Sustainable Development Goals (SDGs). Poverty is one of the most important social determinants of child health, and poverty-reduction programmes have contributed to the decrease of under-five morbidity and mortality in several countries [4,5].

The Brazilian economy experienced one of its strongest economic crises in recent years, with GDP falling by more than 8% since mid-2014 [6,7]. Amid a deep political crisis, a new government came to power on a platform to stabilise the public finances through long-term fiscal austerity measures that will substantially reduce expenditure on social welfare programmes as a percentage of the country’s GDP. The most impactful austerity measure is the Constitutional Amendment 95 (EC95), which will not be limited to the economic crisis, but will last for the next 20 years, reducing the dimension of the already fragile welfare state in Brazil (Box 1) [8,9].

**Box 1. Economic crisis and Brazil’s austerity measures**

**Economic crisis scenarios**

Since 2014, a sharp and deep recession in Brazil has unfolded, with annual GDP contractions of 3.8% and 3.6% in 2015 and 2016, respectively [10]. The economic crisis led to increasing unemployment, mainly in low-income populations, and the poverty rate (those with an income of less than US$43 a month) has increased from 7.4% in 2014 to 8.7% in 2015, with the extreme poverty rate (those with an income of less than US$21 a month) increasing from 2.8% to 3.4%. We model three economic crisis scenarios in our analysis based on 2017 World Bank projections [7]. These estimated that poverty rates for 2016 and 2017 would increase to 9.7% and 9.8%, respectively, in a milder economic crisis (called scenario 1 in the study) and 9.8% and 10.3%, respectively, in a stronger crisis (scenario 2). In scenario 1 the economic crisis would end in 2018, whilst in scenario 2 it would end in 2020. A third economic crisis scenario (scenario 3) was also examined that prolonged scenario 2 until 2022. While the recession technically ended, in terms of GDP growth, in late 2017, the economic crisis persists, with unemployment rates, income inequality, and poverty levels worsening. At the moment of writing, scenario 2 is the most probable: even if GDP grows in 2018, there will be a time lag until the poverty rate starts to decline, considering the high income inequality of the country [7,11].

**Policy response scenarios**

**Fig 1:**
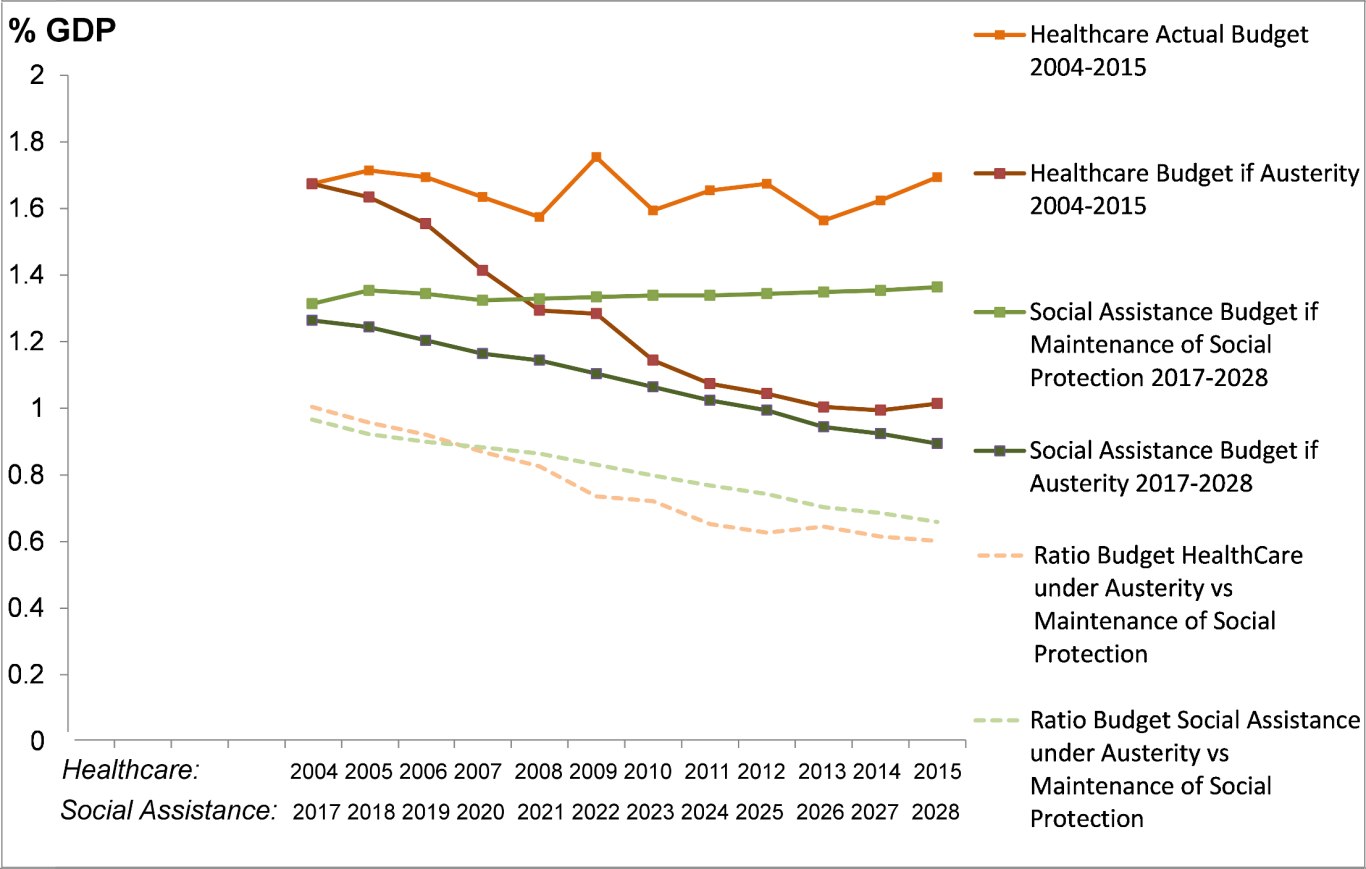
Expenditure on social assistance and healthcare as a percentage of GDP according to the economic crisis and policy response scenarios. For healthcare spending, the comparison is between the real spending in the period 2004–2015 and the simulated spending if the Constitutional Amendment 95 (EC95) were applied during the same period; for social assistance, the comparison is between the simulated spending necessary to maintain the existing levels of protection for the years 2017–2028 and the simulated spending according to the currently implemented EC95.

Since 2016, in the depths of the economic crisis, a newly installed government has initiated a range of fiscal austerity measures [12–14]. The most controversial, and potentially most impactful, was the Constitutional Amendment 95 (EC95), which was approved in 2016 and implemented in 2017 [8,9]. It abolished minimum federal expenditures on social protection and health that were established in the 1988 constitution, and limited the growth in annual federal expenditure on social protection and healthcare to inflation for the next 20 years. Simulations of the effects of EC95 on social assistance and healthcare budgets have been performed by the Brazilian Institute for Applied Economic Research [8,9]. Fig 1 has been drawn based on these data.

Effectively, there is no possibility of real growth in healthcare and social protection expenditures from the federal government, which is important given the current low expenditure on health from public sources (relative to other middle-income countries and countries in Latin America), sizeable predicted population growth in Brazil, and near certain growth in costly health burdens. It is highly likely that the Bolsa Família Programme (BFP) and Estratégia Saúde da Família (ESF) budgets, as major components of the federal health and social protection budget, will be directly affected and their coverage reduced proportionally to EC95 budget reductions (see S1 Text for more detail).

We envision two policy response scenarios: one that follows EC95, with reductions in BFP and ESF coverage (the austerity scenario), and a second hypothetical situation, where funds for the BFP and ESF are increased in line with increases in poverty (a maintenance of social protection scenario).

While economic recession in Brazil technically ended in late 2017, recovery is likely to be fragile given the depth of the economic contraction since 2014 and the ongoing political crisis in the country; continued increases in unemployment, income inequality, and poverty are indicative of a persistent economic crisis [6,7].

The programmes most likely to be affected by austerity measures include the BFP—one of the largest conditional cash transfer programmes of the world—and the ESF—Brazil’s national primary healthcare strategy and principal vehicle for achieving universal health coverage. Available evidence indicates that these programmes have reduced child mortality and health inequalities [4,15,16], and contributed to Brazil’s achievement of Millennium Development Goal 4 (a two-thirds reduction in under-five mortality rate [U5MR]) 2 years early in 2013 (Box 2).

**Box 2. The Bolsa Família Programme and Estratégia Saúde da Família in Brazil**

The BFP was launched in 2003 and expanded quickly. In 2016, it covered 13.6 million families (approximately 25% of the Brazilian population) with a budget of US$8.8 billion—one of the largest conditional cash transfer programmes in the world. Conditional cash transfer programmes are social security systems that provide funds for eligible low-income families, but only if conditions (or conditionalities), usually related to the health and education of their children, are met [5]. The BFP covers extremely poor families (with an income of <US$21 per month) and poor families (with an income between US$21 and US$43) with vulnerable individuals, such as pregnant women, new mothers, children, or adolescents. Monthly cash transfers range from US$18 to US$175 depending on household poverty and the number of vulnerable individuals. Funds are received, when present, by the mother of the family. In the BFP, the conditionalities that must be satisfied include school attendance of children, vaccination and regular health check-ups for children, and prenatal and postnatal visit attendance for expecting and new mothers [17]. Evidence demonstrates that the BFP has improved child nutrition and reduced child mortality in Brazil, with high municipal BFP coverage associated with a 17% reduction in U5MR over the period 2004–2009 [4,18].

**Fig 2:**
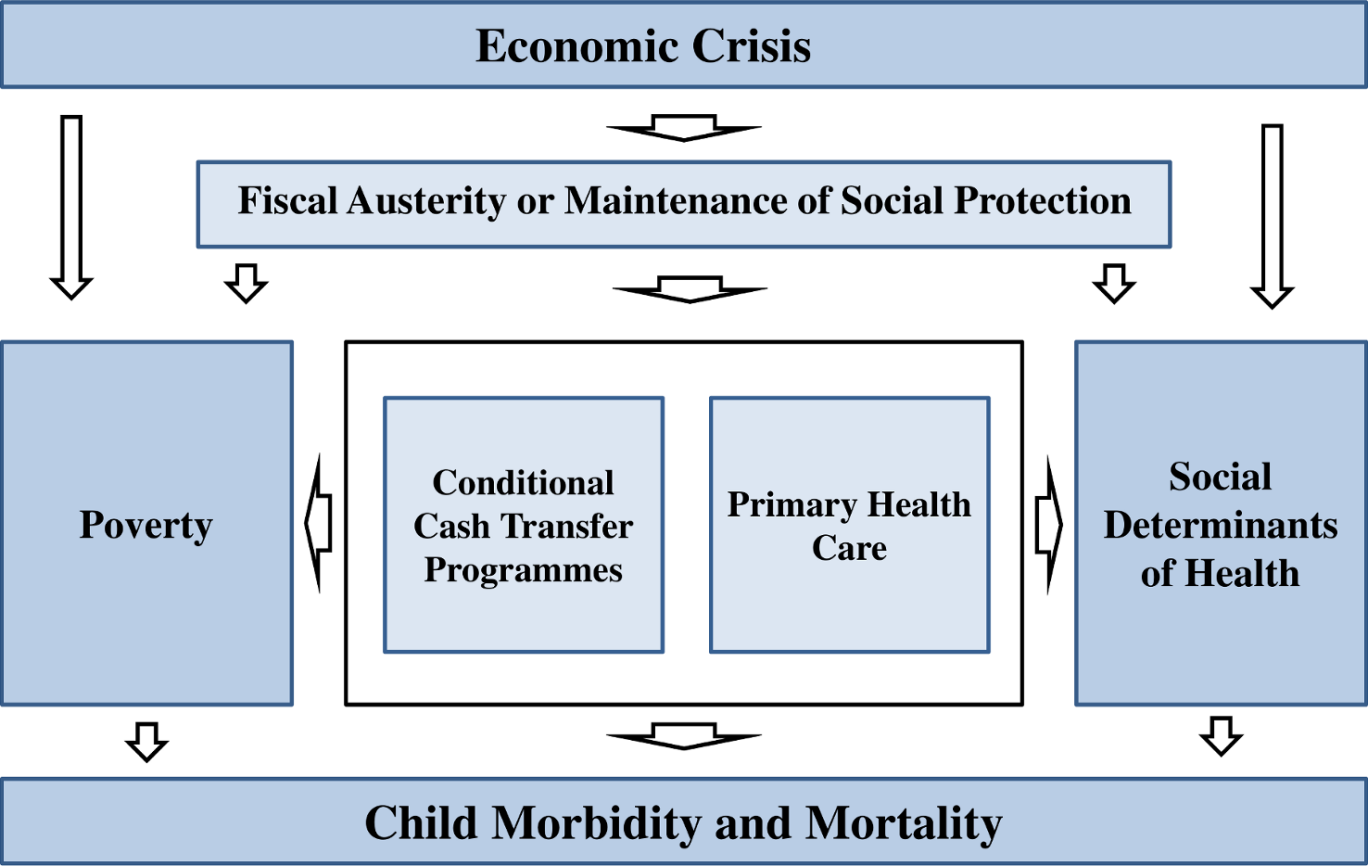
Framework showing pathways between the economic crisis, the austerity and social protection scenarios, conditional cash transfers, primary healthcare, and child health outcomes.

The ESF is a community-based model of primary healthcare, centred on family health teams staffed by a doctor, nurse, nurse assistant, and community health workers providing healthcare to locally defined populations. Approximately 3,500 individuals are registered per team and receive a broad package of primary care services including basic curative care, health promotion, health education, and specific targeted programmes addressing women and children’s health, HIV/AIDS, infectious diseases, and cardiovascular health. A sizeable evidence base has grown demonstrating the impact of the ESF, including on child health. High municipal ESF coverage was associated with a 12% reduction in U5MR over the period 2004–2009 [4]; in addition, there are studies showing that ESF expansion was associated with declines in hospitalisations and mortality from amenable causes [15,16]. A more detailed explanation of how the BFP and ESF affect child health is provided in S2 Text and represented in Fig 2.

Despite an increasing number of retrospective studies on the effects of economic crisis on health outcomes, systematic literature searches yielded no studies forecasting health impacts of economic crisis or austerity measures in LMICs (S1 Text). These types of studies are vital for evidence-based decision-making in these settings. We forecast the effects of the ongoing economic crisis in Brazil on child mortality and hospitalisations during the period 2017–2030 under reductions in BFP and ESF coverage—proportional to the budget constraints of fiscal austerity—or maintenance of the level of social protection currently provided by these programmes. We also evaluate if these reductions will affect Brazil’s progress towards the third and tenth SDGs (improving health and reducing inequalities, respectively). Our forecasts are based on previously documented health impacts of the BFP and ESF using a nationwide cohort of 5,507 Brazilian municipalities [4,15].

## Methods

### Modelling approach and underlying assumptions

This study uses discrete-time microsimulation to forecast the impact of the economic crisis and policy response scenarios in Brazil on overall and cause-specific U5MRs and the under-five hospitalisation rate (U5HR) from 2017 to 2030.

Microsimulation is increasingly used in epidemiology and is particularly useful to evaluate both overall and subgroup impacts of public policies [19–21]. It can provide more accurate estimates of policy effects than traditional compartmental models or time-series forecasting models, which use only the average values in the population. This is because microsimulation allows the modelling of individual-specific characteristics and associated probabilities of the outcome, and when both are derived from existing datasets, the original correlation structure between variables and non-linear effects can be taken into account [21,22]. In our study, we exploited the large number of municipalities in Brazil (using the 5,507 present in 2000 of the 5,570 present today) to deploy a novel ecological-level approach, which both draws on the strengths of microsimulation described above, but additionally captures spillover effects of poverty-alleviation and primary care programmes at the community level [4].

The modelling approach adopted for this study was developed in two stages. First, we created a synthetic cohort of all Brazilian municipalities for the period 2010–2030 as an extension, or post-sample forecasting, of a longitudinal dataset for the period 2000–2010 used previously in retrospective impact evaluations [4,15]. This dataset included all municipalities and all variables. In the previous studies, longitudinal fixed effects multivariate regressions, adjusted for demographic and socioeconomic factors, were used to estimate the effectiveness of the BFP and ESF in decreasing child mortality and hospitalisations. Municipalities were our unit of analysis, and all Brazilian municipalities used the previous study were considered in the study and its sensitivity analyses (*n =* 5,507).

We simulated municipality-specific changes in poverty rates and the other socioeconomic variables over time according to economic crisis scenarios for the years 2010–2030, and BFP and ESF coverage according to policy response scenarios. Changes in relevant demographic variables over time, such as fertility rate and number of live births per municipality, were also modelled.

Second, for each year and each municipality, U5MR and U5HR were estimated as outcomes of the same longitudinal fixed effects regressions using the forecast demographic, socioeconomic, and exposure variables (BFP and ESF coverage) as input values. Fixed effects are used in impact evaluations, both retrospective and forecast, because they include a term to control for unobserved characteristics of the unit of analysis that are constant during the study period, such as some geographical, historical, or sociocultural aspects of each municipality [23]. Mean U5MRs and U5HRs were calculated for the whole country and for subgroups of municipalities. A detailed description of the modelling process and its parameters is provided in S3 Text in accordance with international reporting guidelines (ISPOR-SMDM) [24].

### Data sources

Two types of input data were introduced as parameters in the models (Table 1): the first were municipality-specific demographic and socioeconomic variable values including their municipal time trends, in addition to BFP and ESF coverage values; the second were the effect sizes of all independent variables from regressions on child mortality (overall and from specific causes) and hospitalisations. Demographic and socioeconomic determinants of the U5MRs and U5HRs included in the model were as follows: mean monthly income per capita (Brazilian reais), poverty rate (percentage of individuals with a monthly income of less than US$43), illiteracy rate (of those over 15 years of age), fertility rate, and percentage of the population living in households with adequate sanitation. Values for these variables for the year 2010 were obtained from national census data [25], and values for the years 2011–2030 were extrapolated through exponential decay formulas using the municipality-specific changes over time from the retrospective dataset [4]. For each variable, the parameters of the prediction formula were calibrated comparing the forecast country-level changes over time with the country-level changes over time estimated from the National Household Surveys for the period 2011–2015 [26]. For the two exposure variables, municipal BFP and ESF coverage, values were obtained for 2010–2016 from the Ministry of Health’s Department of Primary Care and Ministry of Social Development [27–29]. Forecast (2017–2030) BFP and ESF coverage data points were simulated according to the economic crisis and policy response scenarios defined below. All variables were modelled as continuous and successively categorised according to cutoffs used in the retrospective evaluation [4], including dimensions of duration of BFP and ESF coverage, as detailed in S3 Text.

**Table 1:** Model inputs and data sources.

Variable	Mean values	Effect sizes (rate ratios)	Data sources of municipal values
Municipal BFP coverage	See Fig 3	See S3 Text, Table A	Ministry of Social Development
Municipal ESF coverage	See Fig 3	See S3 Text, Table A	Datasus, Ministry of Health
Monthly income per capita	See Table 2	See S3 Text, Table A	National census data, IBGE
Poverty rate	See Table 2	See S3 Text, Table A	National census data, IBGE
Illiteracy rate	See Table 2	See S3 Text, Table A	National census data, IBGE
Fertility rate	See Table 2	See S3 Text, Table A	National census data, IBGE
Percentage of the population living in households with adequate sanitation	See Table 2	See S3 Text, Table A	National census data, IBGE

BFP, Bolsa Família Programme; ESF, Estratégia Saúde da Família; IBGE, Instituto Brasileiro de Geografia e Estatística.

### Modelled scenarios: Economic crisis and policy response

We simulated three economic crisis scenarios using poverty rates and mean per capita income from National Household Surveys for the years 2011–2015 and microsimulation from the World Bank for the time periods indicated below [7]. The scenarios considered in this analysis were as follows:

- Economic crisis scenario 1: A milder and shorter economic crisis with a smaller yearly increase (0.55%) in the poverty rate lasting 3 years (from 2015 to 2017)
- Economic crisis scenario 2: A medium economic crisis with a larger yearly increase (0.80%) in the poverty rate sustained over 5 years (from 2015 to 2019), the most probable at the time of writing
- Economic crisis scenario 3: A longer economic crisis with the same percent increase in the poverty rate as scenario 2, but sustained over 7 years (from 2015 to 2021)

In response to the economic crisis, two policy responses were considered in the main analysis, both starting from 2017 (the current year of the study):

- Fiscal austerity: This started to be implemented at the beginning of 2017. Estimates were based on simulations of the impact of the already implemented austerity measures (mainly EC95) on the budget for social protection and healthcare until 2030 [8,9,12]. Reductions in BFP and ESF coverage were modelled as proportional to reductions in the budget.
- Social protection: Maintaining social assistance and healthcare coverage in response to the economic crisis in the period 2017–2030. This response was projected as increases in BFP and ESF coverage proportional to increases in the poverty rate and also, at the end of the economic crisis, decreases in the BFP proportional to poverty reductions and a return to pre-crisis coverage levels for the ESF.

Additionally, a broad range of economic crisis and policy response scenarios were modelled as sensitivity analyses (see S3 Text).

### Simulation of the effects on child morbidity and mortality

Using the 2010–2030 synthetic cohort of covariates and exposure variables (BFP and ESF coverage)—an extension of the retrospective cohort based, as explained above, on real data—for different scenarios, a post-sample forecasting of the U5MRs and U5HRs was performed. Fixed effects predictors were used based on the fixed effects negative binomial regression models in the previous retrospective impact evaluation [4]. In summary, each outcome, for a specific year and specific municipality, was estimated as the product of the fixed effects term of the municipality and the independent variables, with their effects expressed as rate ratios, according to the regression model equation. A detailed explanation of the equations used is provided in S3 Text.

U5MRs for specific causes was modelled based on estimates from the reference retrospective study, which showed a stronger effect of consolidated BFP and ESF coverage (high coverage for at least 4 years) on deaths from malnutrition, diarrhoeal diseases, and lower respiratory infections, than on deaths from other causes, with reductions in municipalities with consolidated coverage of 65%, 53%, and 20%, respectively [4], as detailed in S3 Text.

For each outcome and each scenario, 10,000 simulations were performed using the Monte Carlo sampling method. This allows parameter values to vary in each simulation cycle according to their assumed underlying distribution. Mortality rates were modelled as negative binomial distributions, and other parameters as normal distributions. The effects of the independent variables—expressed as incidence rate ratios (IRRs)—were sampled from normal distributions calibrated with the mean and 95% confidence intervals of the IRR from the retrospective impact evaluation. For each year and each scenario, we obtained a distribution of 10,000 possible values of the outcome (U5MR or U5HR). We estimated the mean and the confidence interval using the 2.5% and 97.5% quantiles from this distribution. CIs are used in microsimulation to represent the uncertainty of the estimates [19,21].

To compare and quantify the expected differences in U5MR and U5HR for our policy response scenarios, we estimated the rate ratios between the two scenarios for each simulation, dividing the U5MR and U5HR in the social protection scenario by the U5MR and U5HR in the fiscal austerity scenario, and obtained the mean IRR with CI for all the simulations. These comparisons were also evaluated in terms of the differences in the total number of deaths and hospitalisations during the study period.

The models were coded and implemented in R version 3.4.0.

### Stratification and inequality analysis

Municipalities were stratified based on quintiles of poverty rate in the baseline year of the simulation (2010), and comparisons between the scenarios described above were performed for each quintile. Changes in U5MR inequalities over time across municipalities were estimated using the U5MR concentration index by municipal poverty rate in the baseline year. Concentration indices are relative measures of inequality that, within health, quantify the gradient of a health outcome across the socioeconomic range. They are well-used measures of inequality as they indicate the extent to which health outcomes are concentrated among the disadvantaged (or the advantaged) [30].

### Calibration and validation of the models

All model parameters were derived from a retrospective impact evaluation [4], the variable of the model that was calibrated was the effect size of the time variable, representing secular changes in U5MR over time. Internal validity of the model was assessed by fitting the fixed effects negative binomial multivariate regression used in the retrospective evaluation [4] to the synthetic dataset created for the period 2010–2030, and verifying that all the obtained coefficients were identical to those introduced as inputs in the model.

External validation of the model was undertaken by comparing the overall national U5MR forecast by our microsimulation with the official Brazilian U5MR estimates during the years 2010–2013 [29]. These were the most accurate and up-to-date data on U5MR available. We estimated the linear regression of predicted versus observed values and the proportion of variance (*R*^2^) explained, obtaining a beta coefficient of 0.95 and an *R*^2^ of 0.98, and we verified that all the observed values where included in the 95% CIs of our simulation. A detailed explanation of the calibration and validation process is available in S3 Text.

### Sensitivity analysis

The robustness of the results was verified through multiple sensitivity analyses. First, we tested the effect of different lengths and intensities of poverty rate increases during our simulated economic crisis scenarios. Differential poverty rate increases according to municipal characteristics at baseline (2010) were also tested, with larger increases in poverty in poorer municipalities and smaller increases in the wealthier municipalities. Second, a broad range of policy responses were tested, including slower reductions in BFP and ESF coverage (less than 4% yearly) under austerity scenarios and heterogeneous BFP and ESF reductions. Third, different values for the effect of the time variable representing secular changes over time were tested. We also evaluated the possibility that the fiscal austerity scenario would be able to reduce and shorten the economic crisis, while the maintenance of social protection would extend the period of economic crisis, comparing the two possible scenarios. All sensitivity analyses are detailed in S3 and S4 Texts.

## Results

### Modelled scenarios: Economic crisis and policy response

Under all economic crisis scenarios, poverty rates are forecast to increase, and income per capita to fall, in the coming years (Table 2). In economic scenario 1 (a milder and shorter crisis), decreases in income and increases in the poverty rate are estimated to cease from 2018 onwards, whilst for scenario 2 (a medium crisis), this occurs 2 years later, in 2020, and in scenario 3 (a longer crisis), in 2022. Other socioeconomic variables included in the analysis follow historical changes over time, showing reductions in illiteracy, declining fertility, and improvements in access to sanitation.

**Table 2:** Mean values of independent variables by economic crisis scenario for 2015–2020, 2025, and 2030.

Economic crisis scenario	Variable	Year							
		2015	2016	2017	2018	2019	2020	2025	2030
**Scenario 1 (shorter crisis)**	Poverty	13.6	15.4	16.8	15.9	15.1	14.4	11.4	9.2
	Income	731.2	720.2	709.5	732.8	755.2	776.8	873.2	952.8
**Scenario 2 (medium crisis)**	Poverty	13.9	15.9	17.4	18.5	19.4	18.4	14.4	11.5
	Income	731.2	720.2	709.5	698.8	688.4	711.0	812.0	895.8
**Scenario 3 (longer crisis)**	Poverty	13.9	15.9	17.4	18.5	19.4	20.2	15.7	12.5
	Income	731.2	720.2	709.5	698.8	688.4	677.7	781.1	866.3
**All scenarios**	Illiteracy	12.3	11.6	11.1	10.5	10.0	9.5	7.6	6.2
	Fertility	1.9	1.8	1.8	1.7	1.7	1.6	1.4	1.3
	Sanitation	91.1	91.9	92.6	93.1	93.5	93.8	94.6	95.0

Income—mean monthly per capita income (Brazilian reais); poverty—percentage of population with an income of less than US$43 per month; illiteracy—percentage of those over 15 years of age who are illiterate; fertility rate—mean children per woman; sanitation—percentage of the population living in households with adequate sanitation.

**Fig 3:**
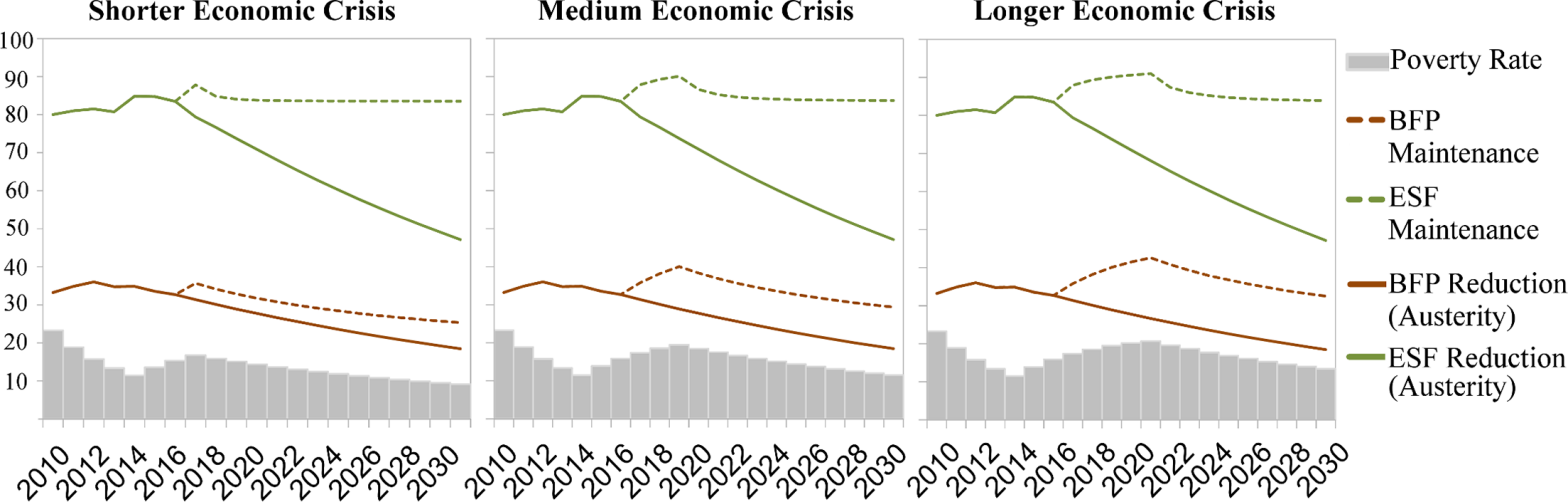
Municipal mean poverty rate, Bolsa Família Programme coverage, and Estratégia de Saúde da Família coverage under different economic crisis and policy response scenarios for 2010–2030. *y*-Axis shows percentage. BFP, Bolsa Família Programme; ESF, Estratégia Saúde da Família.

Fig 3 shows mean poverty rates of all municipalities (from World Bank estimates) under economic scenarios of shorter, medium, and longer crisis and mean municipal BFP and ESF coverage under both policy response scenarios (current fiscal austerity or maintenance of social protection).

**Fig 4:**
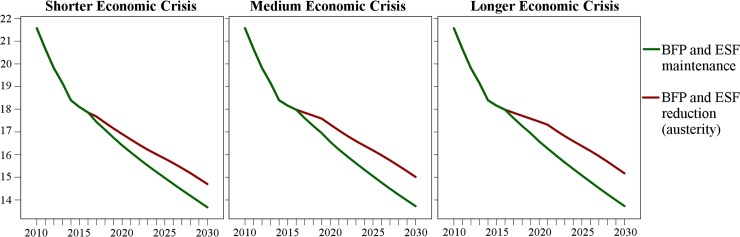
Mean municipal under-five mortality rates (per 1,000 live births) under different economic crisis and policy response scenarios for 2010–2030. BFP, Bolsa Família Programme; ESF, Estratégia Saúde da Família.

Values of all variables in the study period are shown in S3 Text. In all economic crisis and policy response scenarios, mean U5MR is forecast to continue declining, although the magnitude of the reduction varies (Fig 4). In 2015, at the beginning of the economic crisis, we observe a sharp slowing in the annual reduction of the U5MR (0.2% a year) in comparison with the 2010–2015 period (0.8% a year) due to increasing poverty rates.

From 2017 onwards, the two policy responses are associated with different U5MRs (Fig 4). Continuing austerity and the concomitant reductions in ESF and BFP coverage would slow the decline in U5MR relative to maintaining social protection. In the scenario of a milder crisis, which consequently requires smaller increases in BFP and ESF coverage under the maintenance of social protection scenario, the U5MR in 2030 would be 6.98% (95% CI: 5.26%–8.67%) lower compared to fiscal austerity, with 13,954 (95% CI: 4,559–23,418) under-five deaths averted over the period 2017–2030. In the scenario of medium crisis, with maintenance of social protection the U5MR would be 8.57% (95% CI: 6.88%–10.24%) lower in 2030 than under austerity, representing a cumulative 19,732 (95% CI: 10,207–29,285) averted under-five deaths over the period 2017–2030. In the scenario of a longer crisis, the U5MR by 2030 would be 9.53% (95% CI: 7.92%–11.17%) lower with maintenance of social protection compared to the austerity scenario, with 23,424 (95% CI: 13,829–32,880) averted under-five deaths over the study period. Table 3 shows the U5MR rate ratios between the two policy responses for different intensities and duration of economic crisis.

**Table 3:** Under-five mortality rate ratios between the two policy response scenarios by economic crisis scenario for 2015–2030.

Economic crisis scenario	Rate ratio (confidence interval) by year						
	2015	2017	2018	2019	2020	2025	2030
Economic crisis scenario 1 (shorter)	1.000 (0.982–1.018)	0.987 (0.969–1.005)	0.982 (0.964–1.000)	0.976 (0.959–0.994)	0.970 (0.953–0.988)	0.946 (0.929–0.964)	0.930 (0.913–0.947)
Economic crisis scenario 2 (medium)	1.000 (0.982–1.018)	0.987 (0.968–1.005)	0.975 (0.957–0.993)	0.964 (0.946–0.981)	0.956 (0.938–0.973)	0.930 (0.913–0.947)	0.914 (0.898–0.931)
Economic crisis scenario 3 (longer)	1.000 (0.982–1.018)	0.987 (0.968–1.005)	0.975 (0.957–0.993)	0.964 (0.946–0.981)	0.950 (0.932–0.967)	0.921 (0.903–0.937)	0.905 (0.888–0.921)

**Fig 5:**
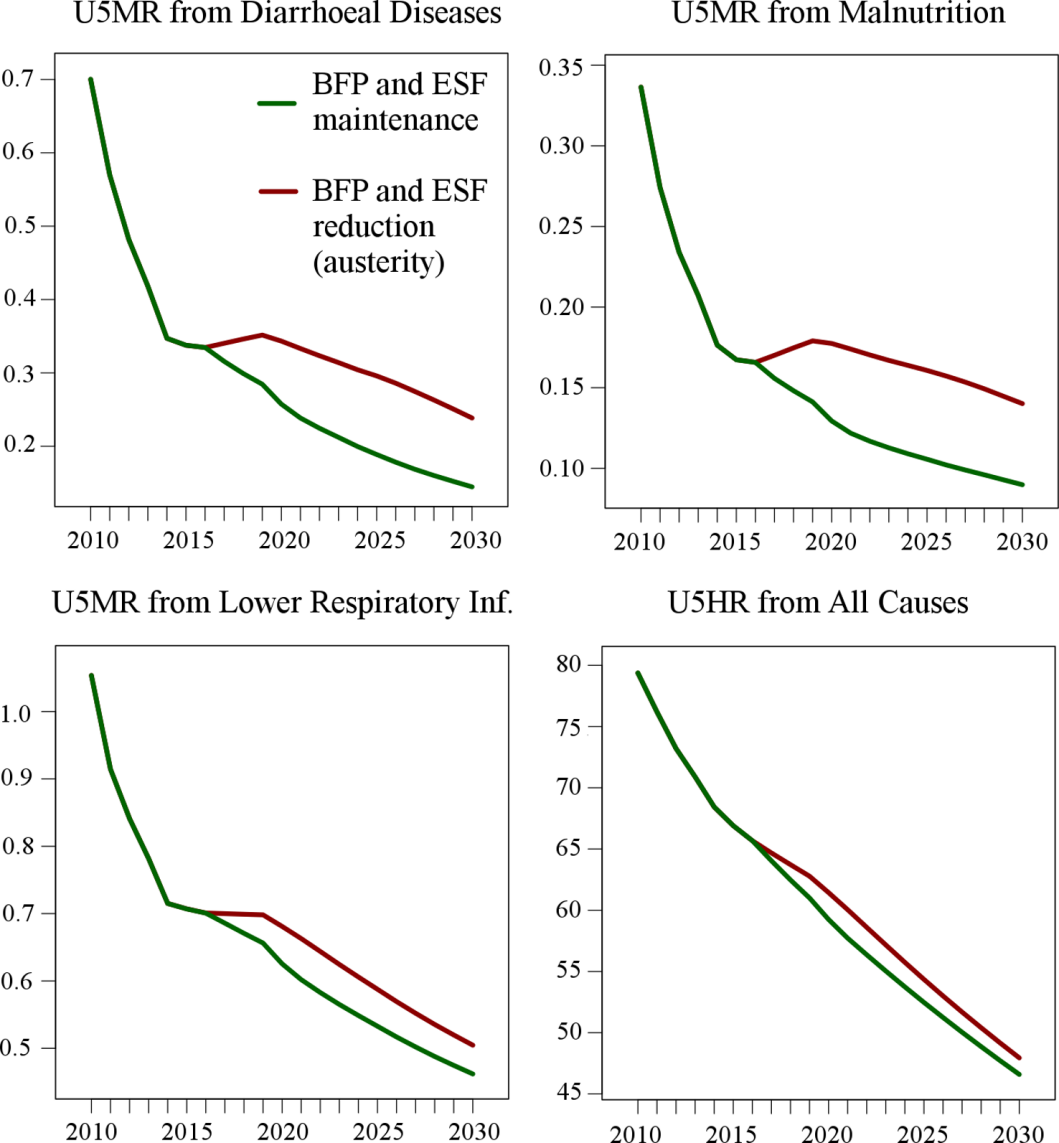
Mean municipal under-five mortality rates (per 1,000 live births) for selected causes and under-five hospitalisation rate (per 1,000 live births) for the period 2010–2030 under the medium economic crisis scenario and for two policy responses (austerity and maintenance of social protection). BFP, Bolsa Família Programme; ESF, Estratégia Saúde da Família; Inf., infection; U5HR, under-five hospitalisation rate; U5MR, under-five mortality rate.

Fig 5 shows forecast U5MRs for diarrhoeal diseases, malnutrition, and lower respiratory tract infections, in addition to overall U5HR, under a medium intensity economic crisis—the most probable at the time of writing (results for scenarios of shorter and longer crisis are detailed in S4 Text).

Attenuations in annual reductions in U5MR and U5HR are observed for both policy responses, but under the austerity scenario, increases in the U5MR are forecast for diarrhoeal diseases and malnutrition. Under the maintenance of social protection scenario, mortality from these causes would continue to decline, albeit at a slower rate, resulting in U5MRs in 2030 that are 39.3% (95% CI: 36.9%–41.8%) and 35.8% (95% CI: 31.5%–39.9%) lower for diarrhoeal diseases and malnutrition, respectively, than under austerity. For lower respiratory tract infections, by 2030 the U5MR would be 8.45% (95% CI: 4.16%–12.01%) lower with maintenance of social protection. The rate ratios for these causes of death are larger than for overall U5MR because the effects of the BFP and ESF were larger in the retrospective analysis (see S3 Text). Annual declines in the U5HR continue under both policy response scenarios, albeit at a slower rate under the austerity scenario. Under the policy response of maintenance of social protection there would be a greater reduction in the U5HR, which by 2030 would be 3.07% (95% CI: 0.99%–5.12%) lower, corresponding to 123,549 (95% CI: 21,248–226,292) averted under-five hospitalisations over the study period 2017–2030.

### Stratification and inequality analysis

**Fig 6:**
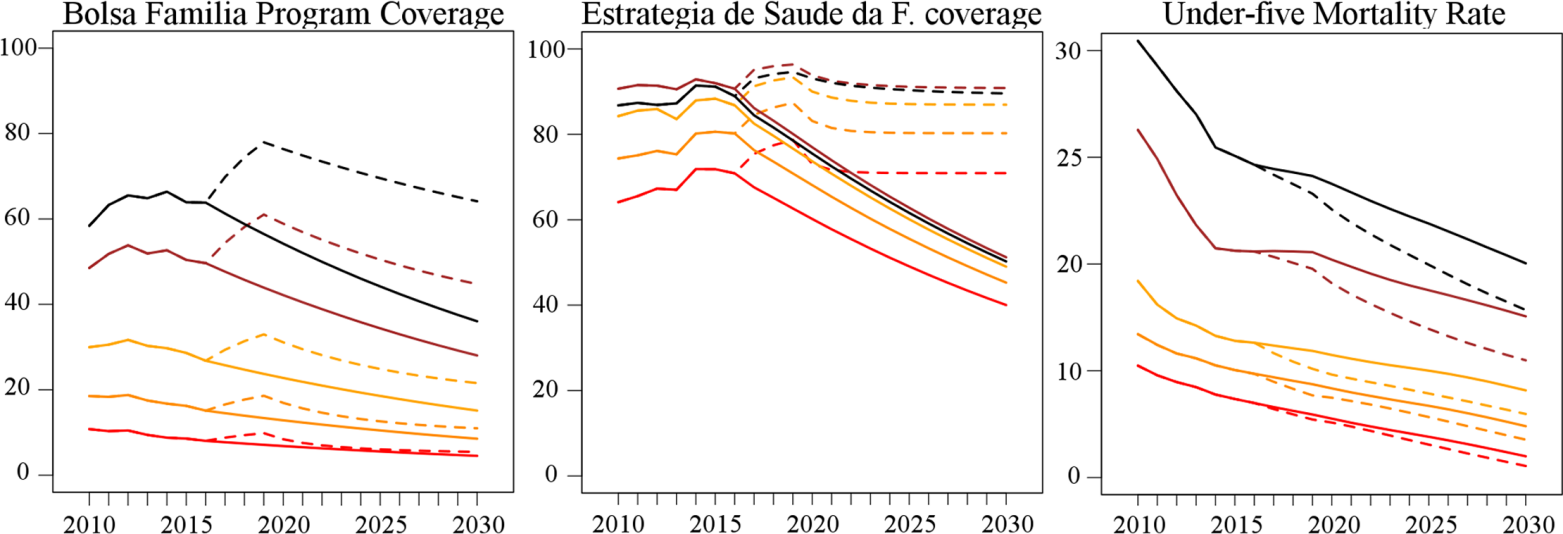
Mean municipal BFP coverage, ESF coverage, and under-five mortality rate (per 1,000 live births) by poverty quintiles of municipalities for 2010–2030 under the medium economic crisis scenario and for both policy responses (austerity and social protection maintenance). BFP and ESF coverage given as percentage. Policy response—fiscal austerity: solid lines; policy response—social protection maintenance: dashed lines; black: first quintile (poorest); brown: second quintile; yellow: third quintile; orange: fourth quintile; red: fifth quintile (richest). BFP, Bolsa Família Programme; ESF, Estratégia Saúde da Família.

Stratification of changes in U5MR by quintiles of municipal-level poverty shows that the effects of the maintenance of social protection are greatest in the poorest municipalities (Fig 6).

Under maintenance of social protection, the U5MR would be 11.01% (95% CI: 7.97%–13.83%) lower than under austerity in 2030 in the first (poorest) quintile (Table 4). This compares to no significant difference (between social protection and austerity) in the fifth (richest) quintile. Concerning inequalities, while the concentration index of U5MR among municipalities is not predicted to decrease under austerity (0.129 [95% CI: 0.122–0.137] in 2015 versus 0.128 [95% CI: 0.121–0.135] in 2030), under the maintenance of social protection it would be 0.111 (95% CI: 0.104–0.119) in 2030, which is 13.3% (95% CI: 5.6%–21.8%) lower than under austerity.

**Table 4:** Under-five mortality rate ratios between the two policy response scenarios in the medium economic crisis scenario by municipality poverty quintile for 2015–2030.

Poverty quintile	Rate ratio (confidence interval) by year			
	2015	2020	2025	2030
Poverty quintile 1	1.000 (0.968–1.033)	0.949 (0.912–0.981)	0.912 (0.882–0.943)	0.890 (0.862–0.920)
Poverty quintile 2	1.000 (0.963–1.038)	0.946 (0.911–0.982)	0.904 (0.871–0.938)	0.883 (0.851–0.916)
Poverty quintile 3	1.000 (0.956–1.046)	0.942 (0.900–0.985)	0.929 (0.888–0.972)	0.921 (0.882–0.964)
Poverty quintile 4	1.000 (0.952–1.048)	0.970 (0.925–1.017)	0.961 (0.915–1.007)	0.950 (0.904–0.998)
Poverty quintile 5	1.000 (0.954–1.049)	0.986 (0.939–1.035)	0.970 (0.924–1.018)	0.959 (0.914–1.007)

### Sensitivity analysis

Results on the U5MRs from diarrhoea, malnutrition, and lower respiratory infections and hospitalisations are similar under the different economic crisis scenarios and have a similar dose–response relationship with the intensity of the crisis as for overall U5MR. Alternative inequality analyses under different economic scenarios also produce similar results. Even if different secular changes in U5MRs over time are introduced in the model, the comparison (in terms of either the IRR or avoided deaths) between scenarios remains unchanged. Sensitivity analyses show that varying the magnitude of BFP and ESF coverage reductions due to austerity measures do not affect our conclusions as all modelled reductions were associated with strong and statistically significant child mortality impacts. Our simulation where fiscal austerity shortened the economic crisis, while the maintenance of social protection extended it until 2030 shows that child mortality would still be 4.43% (95% CI: 2.79%–6.32%) lower in 2030 under the latter scenario.

## Discussion

Our findings show that levels of child mortality in Brazil are likely to be substantially different under a fiscal austerity scenario, modelled as a reduction in coverage of poverty-alleviation and primary care programmes, compared with a scenario where existing levels of social protection of these programmes are maintained and provide coverage of vulnerable populations. Our forecasts indicate that under a scenario that maintains social protection the U5MR would be 8.6% lower in 2030 than under an austerity scenario—with a cumulative impact of almost 20,000 averted under-five deaths over the period 2017–2030. U5MRs from diarrhoeal diseases and malnutrition would be 39.3% and 35.8% lower in 2030, respectively, and there would be 123,000 fewer under-five hospitalisations under the maintenance of social protection. According to our estimates, BFP and ESF coverage reductions would disproportionately impact child mortality in the poorest municipalities and contribute to the persistence of sizeable health inequalities, compromising efforts towards achieving both the third and tenth SDGs.

To our knowledge, this is the first study to evaluate the impact of fiscal austerity measures on health during an ongoing economic crisis in a middle-income country. Similar to Brazil, many other Latin American countries are experiencing economic crises that have stimulated policy-makers to consider fiscal austerity, potentially undermining the longstanding efforts to strengthen their welfare states [31]. A small number of studies in high-income countries have shown mixed results on the impact of economic crises on child health and mortality [2,3]. For example, in Canada and Ireland no effect was detected, but in Spain recession has been shown to affect general health and use of healthcare services in children from vulnerable groups [32]. A combination of economic crisis and externally imposed austerity measures in Greece has been associated with small increases in infant mortality [33]. There is less evidence available from LMICs, although it is plausible that economic shocks may have greater impact in countries where children are more vulnerable and have less social protection than in high-income countries [34]. Approximate estimates for the 2008–2009 crisis in sub-Saharan countries showed an excess of 28,000–50,000 infant deaths [35], while in Bangladesh the prevalence of wasting in children increased 6.7% following the economic crisis [36]. No studies to our knowledge have focused on the impact of austerity measures during an economic crisis in LMICs.

Our prediction of higher mortality from diarrhoea and malnutrition under austerity is consistent with our retrospective evaluation, where the BFP and ESF had a considerably stronger effect on mortality from these causes than on overall U5MR [4] (S3 Text), and with studies showing that these conditions are strongly associated with poverty [37]. Few studies have examined wider, medium-term health system impacts of austerity measures, which are likely to be especially important if disinvestments occur in poverty-alleviation programmes and primary healthcare [38,39]. The predicted health impacts of austerity through the BFP and ESF coverage reductions may occur through multiple ways and multiple mechanisms. Evidence shows that the BFP improves both the quality and quantity of food for poor families [40], leading to better nutritional status of children in the poorest regions of Brazil [18], and likely explaining reductions in U5MR [4,41]. The BFP also improves child health more directly by promoting prenatal care for pregnant women and vaccination coverage and routine check-ups for children as key conditions that must be fulfilled to receive funds [4,41]. Regarding the ESF, evidence suggests that programme expansion reduces child mortality by increasing access to prenatal care and improving vaccination coverage [15,42]. Furthermore, the ESF increases the number of basic medical activities and domiciliary visits in its area of coverage [15], and also reduces inequalities by exerting a greater impact in municipalities with lower Human Development Index [42].

The main strength of this study is the use of a synthetic cohort of 5,507 municipalities built as an extension of a pre-existing 10-year retrospective cohort used in previous impact evaluations [4,15]. This synthetic cohort is able to incorporate the real correlation structure between variables and to model real municipality-specific parameters and changes in municipal-specific variables over time, which have been calibrated with national-level data. The external validation of the models—using a different data source than the one used for the calibration process—is another strength of the study, together with the extensive sensitivity analyses performed (S3 and S4 Texts). The use of estimates of programme effectiveness coming from the same country under study is an important advantage of the models, given that similar public policies could be implemented differently and have different effectiveness in other countries [23,39].

There are pertinent limitations of the study, mainly stemming from uncertainty around the future macroeconomic scenarios in Brazil. This is due to the current unstable political and economic situation, creating uncertainty around the forecasting of poverty rates, income, and the other independent variables. Therefore, additional scenarios were forecast in sensitivity analyses, with all—even simulation of different intensities and lengths of the economic crisis—showing that the differences between the two policy scenarios (long-term austerity versus maintenance of social protection) remain large and of comparable magnitude (S4 Text). We modelled the impact of austerity on BFP and ESF coverage as there is strong evidence these policies confer large protective effects for child morbidity and mortality [4,15,41,42], but this study is also limited in that austerity may affect other government policies and parts of the economy we have not considered. It is likely our estimates of the negative impact of austerity on child health are conservative as they do not reflect public spending constraints in other areas, e.g., education, housing, and other welfare programmes [8,9,12]. Another limitation of the study is our modelling of policy effectiveness at an ecological rather than individual level. Studies evaluating mortality impacts of public policy using individual-level data are uncommon—mainly due to the rarity of the outcome and the extensive follow-up time required. Additionally, individual-level studies are not able to evaluate important spillover effects of poverty-alleviation programmes in the community [4,43]. We used a measurement of the intensity of the intervention (coverage of the BFP and ESF) specifically linked to the population group most vulnerable to poverty-related mortality, thus reducing the possibility of changes in mortality being derived from non-exposed populations [4]. Impact evaluations using ecological panel data have been extensively used to evaluate the effectiveness of country-wide, and often heterogeneous, implementations of public policies in Brazil, and achieve higher external validity than individual-level studies [4,15,41,42,44]. Our findings show that, even if we model that austerity measures (EC95) will shorten the duration of the economic crisis in Brazil, its medium and long-term effects on child mortality remain strongly detrimental.

Brazil has implemented bold policies to reduce poverty and achieve universal health coverage over the past 20 years [45,46], with two-thirds of the population now covered by the ESF and one-fourth by the BFP [4,15,27,28]. These policies have contributed to important improvements in health outcomes and have decreased health inequalities [46]. Our findings indicate that the current economic crisis together with the package of austerity measures may jeopardise further gains in poverty reduction and improvements in health outcomes, especially among poor Brazilians. Ongoing monitoring of the poverty and health impacts of austerity policies in Brazil and the extent to which these constrain achievement of key SDGs will afford important learning for policy-makers globally.

In conclusion, the results of our study show that implementation of fiscal austerity measures could contribute to a large number of preventable child deaths and hospitalisations in Brazil, threatening attainment of the SDGs related to child health and inequalities.

## Supporting information

S1 Text Simulations of fiscal austerity. (DOCX)

S2 Text Mechanisms of effectiveness of the Bolsa Família Programme and Estratégia Saúde da Família. (DOCX)

S3 Text Detailed description of the modelling process according to international model reporting guidelines (ISPOR-SMDM). (DOCX)

S4 Text The 3 economic crisis scenarios: Comparative results. (DOCX)

## Abbreviations

BFP: Bolsa Família Programme
EC95: Constitutional Amendment 95
ESF: Estratégia Saúde da Família
IRR: incidence rate ratio
LMICs: low- and middle-income countries
SDGs: Sustainable Development Goals
U5HR: under-five hospitalisation rate
U5MR: under-five mortality rate
