# Supplementary material for: Child morbidity and mortality associated with alternative policy responses to the economic crisis in Brazil: A nationwide microsimulation study
Source: PLoS Med. 2018 May 22;15(5):e1002570. doi: 10.1371/journal.pmed.1002570 (PMC5963760; doi:10.1371/journal.pmed.1002570)
Supplement: S1 Text — (DOCX) [file pmed.1002570.s001.docx]

**S1 Text. Literature Review and Simulations of Fiscal Austerity**

**A. Literature Review**

We searched MEDLINE and Latin American literature databases (LILACS) for any study on the impact of economic crisis and austerity on child health outcomes published before September 1, 2017, with the terms “economic crisis/austerity”(and related) and ”children/under-five"(and related), and for articles in the reference list of the selected papers.

We also conducted a more focused search on forecasting of economic crisis and austerity measures, using the terms “economic crisis/austerity”(and related) and ”forecasting"(and related).

A small number of studies in high income countries have shown mixed results on the impact of economic crises on child health and mortality. There is less evidence available from LMICs, with some estimates of effects of the 2008-2009 crisis in Sub-Saharan countries and on child malnutrition in Bangladesh. No studies have focused on the impact of austerity measures during an economic recession in LMICs, and no studies undertook prospective forecasting.

All relevant references have been presented in the discussion section.

**B. Simulations of Fiscal Austerity**

Several simulations have been run on how fiscal austerity could affect the coverage of the Bolsa Familia Programme (BFP) and the Estratégia Saúde da Família (ESF).

The most probable scenarios have been drawn from two technical notes from the governmental Institute of Applied Economic Research (IPEA), which estimated the impact of the *Emenda Constitucional 95* (EC95) on the budget for Social Assistance policies [1] and for the National Health System [2] respectively.

EC95 limits yearly Federal Expenses for Social Assistance (SA) and Healthcare for the period 2017-2037 at the values of the previous year increased for inflation, so while it will slightly increase the budget each year, this increase would be insufficient to maintain present levels of services offered to the population [3]. This will be due to 3 main reasons:

1. Population growth, which will be approximately 1% per year in the next years [1], reducing the amount of money per capita available both in SA and healthcare.

2. Population ageing, which in the Social Protection sector will increase budget needs for retirement programmes - mainly Beneficio de Prestacao Continuada (BPC) - restricting resources for other programmes, including BFP. It has to be considered which BPC and BFP account for 56% and 36% of the total Ministry of Social Development budget respectively [1].

3. Higher yearly costs for drugs and medical supplies and incorporation of new drugs and technologies, which will increase per capita expenses for healthcare [4].

Simulations of the budget necessary to maintain constant the levels of SA in the period 2017-2037, considering the factors above mentioned, versus the budget which will be provided under the EC95 have been performed in the IPEA technical note and are shown in Figure A.

For the healthcare system, or Unified Health System (Sistema Único de Saúde, SUS), in the same figure the hypothetical effects of the EC95, if it was implemented in the period 2000-2015 - have been compared to the budget which has been applied during the same period and which allowed the maintenance and some expansions of healthcare services [2]. It has to be considered that the SUS has suffered from chronic underfunding since its creation [5].

The yearly ratios between EC95 and non-EC95 budgets have been calculated, and they have been fitted with an exponential decay formula obtaining an estimate of the percent change. While for the Social Assistance Budget the estimated percent decrease was 4% yearly, for healthcare this was estimated to be 5%.Considering these last estimates where from a period of expansion of coverage of the healthcare system, which is not reasonably expected to happen in the next years, for this microsimulation exercise of austerity scenarios a percent decrease rate of 4% both for BFP and ESF have been applied.

**Fig A. Expenditure as percentage of GDP on social assistance and healthcare according to the economic austerity and maintain social protection scenarios.**


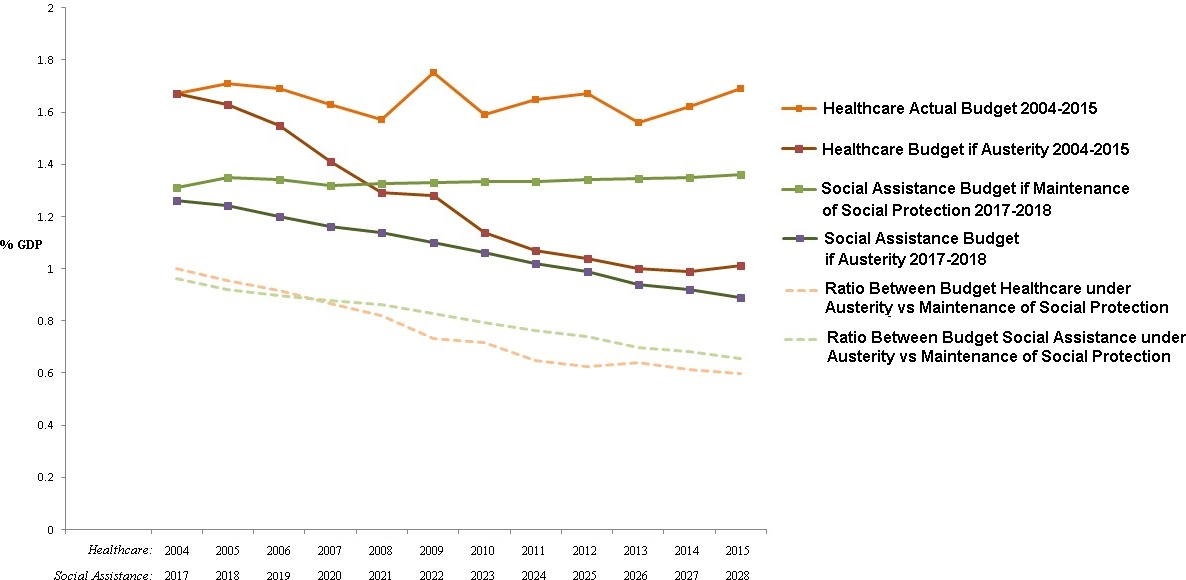


***For healthcare spending, the comparison is between the real spending in the period 2004-2015 and the simulated spending if EC95 was applied during the same period, for social assistance the comparison is between the simulated spending necessary to maintain its existing levels of protection for the years 2017-2028 and the simulated spending according to the currently implemented EC95.***

Austerity measures are likely to affect a broad range of benefits and services offered under Social Assistance programmes and through the Health Care System. For this microsimulation exercise only BFP and ESF impacts were modelled as there is robust evidence that these programmes confer protection against child mortality.

In the case of BFP a restriction in the budget will be more plausibly converted into a reduction of BFP municipal coverage through better targeting (reducing inclusion errors) and deregistering of families that become ineligible. As explained elsewhere [6], BFP municipal coverage better represents BFP effects than BFP coverage of the eligible poor in the models because the former takes account of inclusion errors, which are for the vast majority individuals slightly above the income per capita eligibility threshold and subsequently have a similar health vulnerability. Municipal BFP coverage also includes spill-over effects of the money allowances, in particular for poor municipalities where BFP represent an important part of the local GDP [7,8].

In 2017, a reduction in the number of BFP beneficiaries has already happened: a simple preliminary calculation using official data gathered from Matriz de Informação Social has been performed, comparing July 2016 with the values of July 2017, finding in this initial period a reduction of coverage (8.37%) and nominal spending (8.74%) [9].

In the case of ESF, restrictions in the budget would convert into reductions of coverage primarily because human resources represent a great part of ESF costs [10-12]: Lower health financing would likely produce inactivation of some ESF units or leave ESF teams working without the recommended number or composition of health professionals [13-15].

**S1 Text References**

1. Paiva AB, Mesquita ACS, Jaccoud L, Passos L. [The new tax regime and its implications for social assistance policy in Brazil.] [Portuguese]. Technical Note No.27. Brasilia, Brazil: Instituto de Pesquisa Econômica Aplicada (IPEA), 2016.
2. Vieira FS, Benevides RPdSe. [The impacts of the New Tax Regime for the financing of the Unified Health System and for the realization of the right to health in Brazill] [Portuguese]. [Portuguese] Technical Note No.28. Brasilia, Brazil: Instituto de Pesquisa Econômica Aplicada (IPEA), 2016.
3. Rossi P, Dweck E. Impacts of the new fiscal regime on health and education. Cadernos de saude publica 2016; 32:12-15.
4. Ocké-Reis CO, Marmor TR. The Brazilian national health system: an unfulfilled promise? Int J Health Plann Manage. 2010;25(4):318-29.
5. Ocké-Reis CO, Souza CS. [Regulation of the prices of individual health plans]. [Portuguese] Rev. Econ. Polit. 2011, 31:455-470.
6. Rasella D, Aquino R, Santos CAT, Paes-Sousa R, Barreto ML. Effect of a conditional cash transfer programme on childhood mortality: a nationwide analysis of Brazilian municipalities. The Lancet 2013; 382(9886): 57-64.
7. Duarte GB, Sampaio B, Sampaio Y. [Bolsa Família Program: impact of transfers on food expenses in rural families]. [Portuguese] Rev. Econ. Sociol. Rural 2009, 47(4):24-29.
8. Barros AR, Athias D. [Minimum wage, Bolsa Família and recent relative performance of the Northeast] [Portuguese]. Revista de Economia Política, 2013, 33(1):179-199.
9. Ministerio do Desenvolvimento Social e Combate a Fome. Matriz de Informação Social. 2017. http://aplicacoes.mds.gov.br/sagi/mi2007/tabelas/mi_social.php (accessed 08/04 2017).
10. Marques R, Mendes A.[The financing of Basic Health Care and the Family Health Strategy in the Unified Health System]. [Portuguese] Saúde Debate, 2014, 38(103):900-916.
11. Viera RS, Servo LMS. [Estimates of human resources costs in basic care: Family health teams (ESF) and Oral Health Teams (ESB). Research Need to Fund Basic Care]: [Portuguese] Ipea: Disoc, 2013. Mimeo. (Nota Técnica Brasília, 16).
12. Ruiz D. [Implantation of the National Policy of Basic Attention and of the National Policy of Health Promotion in the year 2016]. [Portuguese] Observatório de Análise Política em Saúde. Instituto de Saúde Coletiva – UFBA, 2017.
13. Rossi P, Dweck E. Impacts of the new fiscal regime on health and education. *Cadernos de saude publica* 2016; 32:12-15.
14. de Souza LEPF. The right to health in Brazil: A Constitutional guarantee threatened by fiscal austerity. J Public Health Policy. 2017 Nov;38(4):493-502.
15. Doniec K, Dall'Alba R, King L. Austerity threatens universal health coverage in Brazil. . Lancet. 2016 Aug 27;388(10047):867-8.
