# Supplementary material for: Child morbidity and mortality associated with alternative policy responses to the economic crisis in Brazil: A nationwide microsimulation study
Source: PLoS Med. 2018 May 22;15(5):e1002570. doi: 10.1371/journal.pmed.1002570 (PMC5963760; doi:10.1371/journal.pmed.1002570)
Supplement: S2 Text — (DOCX) [file pmed.1002570.s002.docx]

**S2 Text. Mechanisms of Effectiveness of Bolsa Familia Program and Estrategia de Saude da Familia**

A detailed description of the causal pathways between poverty, BFP and ESF, and child morbidity and mortality - used for the development of the previous retrospective impact evaluation - is provided elsewhere [1]. Here we provide an overall theoretical framework for the model. A wealth of evidence highlights the relationship between the Bolsa Familia Program (BFP) and Estrategia de Saude da Familia (ESF), and child health outcomes, with a range of potential explanatory pathways. Regarding the BFP, the money received by the household most likely reduces the household poverty burden and improves living conditions [2]. This can additionally remove or reduce potential barriers to accessing health care (for example work commitments or transportation costs), not only for children, but also the rest of the family. A second explanatory pathway between the BFP and child survival may be mediated through improvements in child health directly from the specific health conditionalities [3]. These include usage of prenatal care, postnatal care, and health and nutrition education activities for mothers, in addition to a regular vaccination schedule and routine check-ups for growth and development for children younger under 7 years old. Evidence shows the BFP has increased prenatal care and vaccination coverage [1], which are effective interventions for prevention of child mortality. Maternal education is one of the strongest determinants of child health, which are likely to be mediated through better nutrition, hygiene practices, and care-seeking behaviors when ill. Child growth monitoring, another BFP conditionality, may constitute a third explanatory pathway as it can provide an entry point for preventive and curative health-care services and can increase contact with the health system [4]. This is likely given access to healthcare is an important determinant of child survival in LMICs [3,5].

As a primary healthcare service, there are multiple ways through which the ESF can bring about improvements in health outcomes. ESF teams are responsible for registering and delivering healthcare services to defined local populations. The range of services should be comprehensive and are care free of charge. Firstly, expanded access to curative services may impart improvements in health outcomes for children. Child and maternal health services are identified as an essential service that must be delivered by ESF teams. Many causes of child deaths (e.g. intestinal infections or vaccine preventable conditions) may be easily preventable by access to basic services. Secondly, community health workers may play an important role in expanding access to health services through outreach and educating mothers and families about potential services and ways of access. Thirdly, health promotion, prevention and educational activities provided by ESF teams may have a more indirect mechanism of action in improving health literacy of enrolled population, preventing uptake of riskier behaviors and exacerbation of existing risk factors and encouraging healthier behaviors.

**Fig A: Model of the mechanisms of effectiveness of BFP and ESF on child morbidity and mortality.**

**
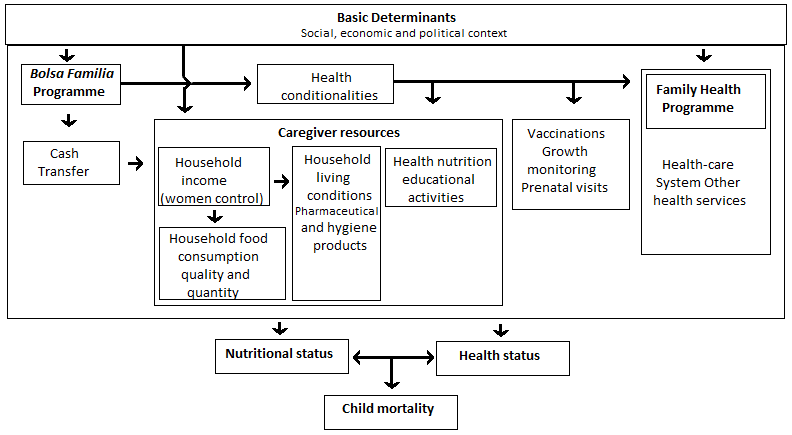
**

**S2 Text References**

1. Rasella D, Aquino R, Santos CAT, Paes-Sousa R, Barreto ML. Effect of a conditional cash transfer programme on childhood mortality: a nationwide analysis of Brazilian municipalities. The Lancet 2013; 382(9886): 57-64.

2. Ranganathan M, Lagarde M. Promoting healthy behaviours and improving health outcomes in low and middle income countries: a review of the impact of conditional cash transfer programmes. Prev Med 2012; 55 (suppl): S95–105

3. Gaarder MM, Glassmanb A, Todd JE. Conditional cash transfers and health: unpacking the causal chain. J Develop Eff ect 2010; 2: 6–50

4. Guanais FC. The combined effects of the expansion of primary health care and conditional cash transfers on infant mortality in Brazil, 1998-2010.Am J Public Health. 2015 Oct;105 Suppl 4:S593-9, S585-92.

5. Lagarde M, Haines A, Palmer N. The impact of conditional cash transfers on health outcomes and use of health services in low and middle income countries. Cochrane Database Syst Rev 2009; 4: CD008137
