# Supplementary material for: Child morbidity and mortality associated with alternative policy responses to the economic crisis in Brazil: A nationwide microsimulation study
Source: PLoS Med. 2018 May 22;15(5):e1002570. doi: 10.1371/journal.pmed.1002570 (PMC5963760; doi:10.1371/journal.pmed.1002570)
Supplement: S3 Text — (DOCX) [file pmed.1002570.s003.docx]

**S3 Text. Detailed description of the modeling process**

The following section of the webappendix text provides details of each of these processes in accordance with standard international modelling reporting guidelines (ISPOR-SMDM) (Caro, 2012).

The modelling approach adopted for this study was developed based on two stages.

In the first stage, we created a synthetic cohort of all Brazilian municipalities for the period 2010-2030 as an extension of a longitudinal dataset for 2000-2009 used in a previous ex-post impact evaluations performed by the authors,^1,2^ where fixed effects multivariate regressions, adjusted for demographic and socioeconomic factors, were used to estimate the effectiveness of the BFP and ESF on child hospitalizations and mortality. We simulated municipality-specific trends for poverty rates and the other demographic and socioeconomic variables according to economic crisis scenarios for the years 2010-2030, and BFP or ESF coverage according to social protection policy response scenarios options.

In the second stage, for each year and each municipality, U5MRs and U5HRs were estimated as outcome of the same multivariate fixed effects regressions using the forecast demographic, socioeconomic and exposure variables (BFP and ESF coverage) as input values. Mean U5MRs and U5HRs were calculated for the whole country and for subgroups of municipalities.

**Purpose of the Model and its Applications**

The developed model had the overall purpose to simulate the effects of socioeconomic and policy coverage changes on health outcomes in Brazil using ecologic-level data and - when available - retrospective ecologic datasets. Elements of flexibility have been introduced in the code to allow simulation of different sets of variables and different regression models.

**Theoretical Framework of the Model**

See S2 Text.

**Data Sources, Inputs, outputs, and other parameters**

Two types of input data were introduced as parameters in the models: the first were municipality-specific demographic and socioeconomic variables values-including their trends plus BFP and ESF coverage values; the second were the effect sizes of all the regression independent variables on child mortality, overall and from specific causes, and hospitalizations. Demographic and socioeconomic determinants of U5MR and U5HR employed in the model were: mean monthly income per capita (R$), poverty rate (individuals with an income of less than R$140), illiteracy rate (of those over 15 years of age), fertility rates (mean children per women), and percentage of the population living in households with inadequate sanitation. Values for these variables for the year 2010 were obtained from National Census data (IBGE 2017)^3^ and values for the years 2011-2030 were extrapolated. For each variable the municipality-specific extrapolation was performed through exponential decay formulas using the municipality-specific time trends obtained from the retrospective dataset in 2000-2010. Apart from the trends of poverty rate showed in the core manuscript, only income per capita was modelled as affected by economic crisis according to the three proposed scenarios (Figure A and Figure B). The trends of the other determinants of child mortality has been modelled as not affected by the economic crisis and their specific trends where obtained - for each municipality - from the linear trend of the decade 2000-2010 expressed as percent decrease for the year 2010 an inserted in the percent decrease/exponential decay formula:

*V_it_ = V_i_ (1-P_i_)^Kt^*

*V_it=_*Value of the variable for the municipality i at the time t

*V_i=_* Value of the variable for the municipality i at the time 0 (year 2010).

*t*= Time

*P_i_*_=_ Percent decrease of the municipality i (obtained for the period 2000-2010)

*K*=Calibration term

For each municipality and each variable trends for the period 2010-2030 have been estimated according to the above formula, and they have been calibrated (varying the term K) with the real variable values at the country level for the years 2011-2014 obtained from the National Household Surveys (PNAD).^3^

**Fig A. Box Plots of the trends of the municipal values of the independent variables not affected by the economic crisis in the period 2010-2030.**

*
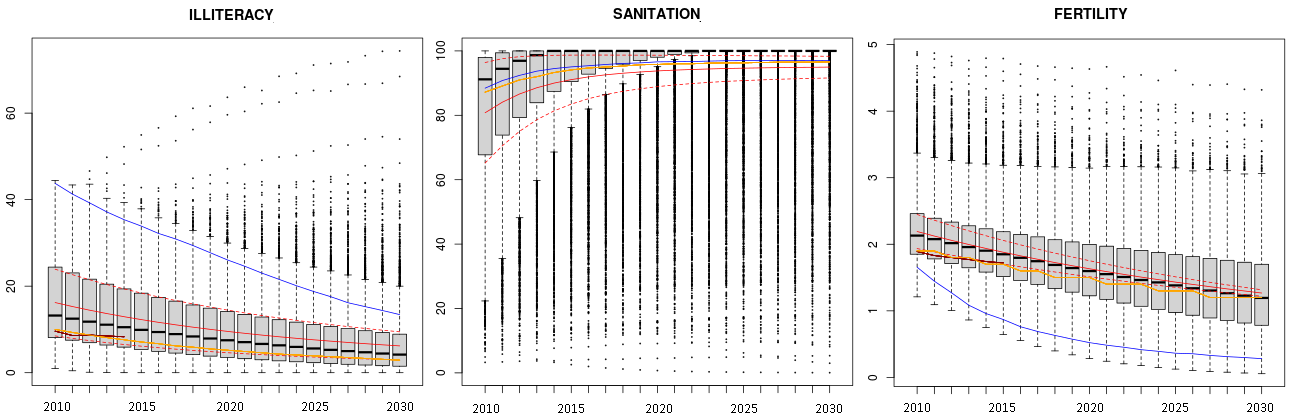
*

**BFP and FHP coverage**

For the two exposure variables, BFP and ESF coverage, values were obtained -for the period 2010-2016- from the Ministry of Health’s Department of Primary Care and Ministry of Social Development.^4,5^ Forecast (2017-2030) BFP and ESF coverage data points were simulated according to the different scenarios defined below. All variables were modelled as continuous and successively categorized according to cut-offs used in the retrospective evaluation, including dimensions of duration of BFP and ESF coverage.^1^

The categorization of the coverage of the Bolsa Familia Program and the Estrategia de Saude da Familia were performed based on the previous ex-ante evaluation study.^1^ For the BFP, it is possible to conceive of two indicators of coverage. The first is coverage of the target population, calculated as the number of families enrolled in the BFP in a municipality divided by the number of eligible families (according to BFP criteria) in the same municipality. The second is coverage of the total population, calculated as the number of individuals enrolled in the BFP (obtained by multiplying the number of beneficiary families by the average family size) divided by the total population of the same municipality. The coverage indicator used in the study combine both indicators. The categories for this BFP coverage were: low (BFP coverage of the total population of the municipality from 0.0% to 17.1%), intermediate (17.2–32.0%), high (>32.0%), and consolidated (BFP coverage of the total population of the municipality >32.0% and, at the same time, BFP coverage of the target population ≥100% for at least the previous 4 years). The cutoffs used for the categorization (17.1% and 32.0%) represented the terciles of the distribution of BFP coverage of the total population. This indicator, adjusted in the models for the percentage of the target population in the municipality, capture both the effect of programme duration and of possible programme externalities (ie, positive spillover effects on programme-ineligible inhabitants) in the municipality. Yearly coverage of the FHP can be calculated as the ratio of the total number of individuals registered in this programme to the population of the municipality, and it was categorized, for comparability reasons, as in previous studies: without FHP, incipient (<30.0% of the population), intermediate (30.0–69.9% or ≥70.0% for less than the previous 4 years), and consolidated (≥70.0% for at least the previous 4 years)

**Fig B. Box Plots of the trends of the municipal values of the independent variables affected by the economic crisis and austerity/social protection strengthening measures in the period 2010-2030.**


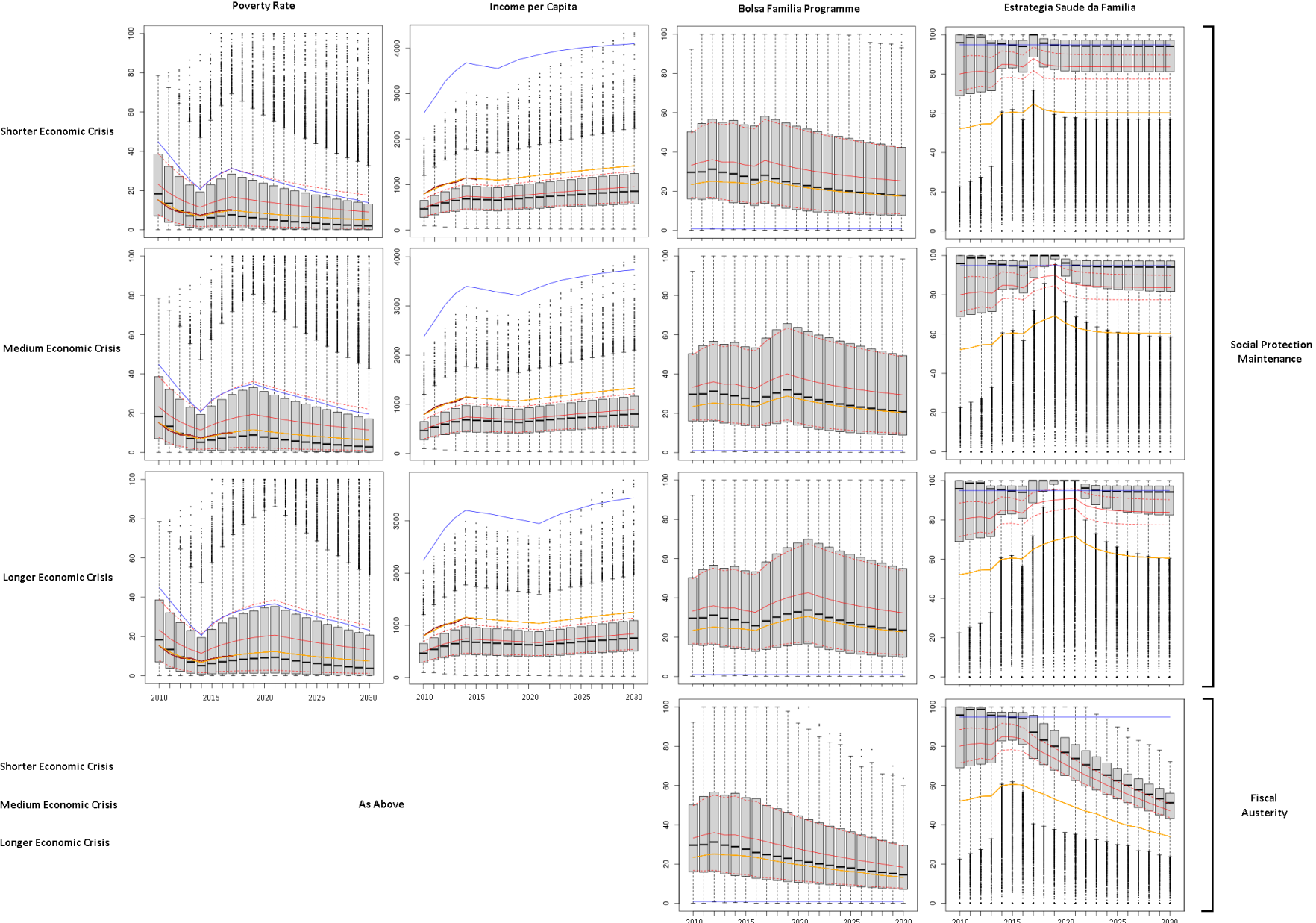


**Effectiveness Parameters of the Model**

The effect size - expressed in terms of Rate Ratios between levels of the categorical variables - of each variable in each specific outcome come from a previous retrospective impact evaluation^1^ and are presented in Table A. Thresholds for the categorization of variables are in parentheses.

**Table A. Mortality Rate Ratios expressing the association between dependent variables and the outcomes from the ex-post impact evaluation of reference for the modelling study.**

|  | **Overall Under-five Mortality** | **U5MR from Diarhoeal diseases** | **U5MR from Malnutrition** | **U5MR from Lower respiratory infections** | **Overall Under-five hospitalization Rate** |
| --- | --- | --- | --- | --- | --- |
| **BFP Population Coverage** |  |  |  |  |  |
| **Low (0.0-17.1%)** | 1.00 | 1.00 | 1.00 | 1.00 | 1.00 |
| **Intermediate (17.2-32.0%)** | 0.94 (0.92-0.96) | 0.83 (0.74-0.92) | 0.66 (0.57-0.77) | 0.96 (0.88-1.05) | 0.96 (0.95-0.97) |
| **High (>32.0%)** | 0.88 (0.85-0.91) | 0.68 (0.59-0.80) | 0.54 (0.44-0.67) | 0.94 (0.82-1.07) | 0.92 (0.90-0.94) |
| **Consolidate (>32.0% and TPC ≥100% for**  **at least 4 years)** | 0.83 (0.79-0.88) | 0.47 (0.37-0.61) | 0.35 (0.24-0.50) | 0.80 (0.64-0.99) | 0.84 (0.81-0.86) |
| **FHP municipality population coverage** |  |  |  |  |  |
| **No FHP (0.0%)** | 1.00 | 1.00 | 1.00 | 1.00 | 1.00 |
| **Incipient (<30%)** | 0.99 (0.94-1.04) | 0.90 (0.67-1.17) | 0.88 (0.60-1.29) | 0.83 (0.68-1.00) | 0.93 (0.91-0.96) |
| **Intermediate (≥30%)** | 0.93 (0.88-0.97) | 0.71 (0.54-0.93) | 0.72 (0.49-1.07) | 0.71 (0.58-0.86) | 0.97 (0.94-1.00) |
| **Consolidated (≥70% and implemented for**  **at least 4 years)** | 0.88 (0.83-0.93) | 0.53 (0.39-0.71) | 0.59 (0.39-0.91) | 0.70 (0.56-0.87) | 0.99 (0.96-1.03) |
| **Income per person (monthly, >BR$380)*** | 0.95 (0.92-0.97) | 0.79 (0.67-0.94) | 0.79 (0.67-0.94) | 0.83 (0.73-0.94) | 0.97 (0.95-0.99) |
| **Proportion of municipality population eligible for BFP* >22.4%** | 1.07 (1.03-1.12) | 1.36 (1.09-1.69) | 1.36 (1.09-1.69) | 1.20 (1.01-1.47) | 0.99 (0.97-1.02) |
| **Proportion of individuals living in households with inadequate sanitation* <16.7%** | 1.10 (1.05-1.15) | 1.29 (1.05-1.71) | 1.29 (1.05-1.71) | 1.09 (0.90-1.31) | 1.11 (1.09-1.15) |
| **Proportion of individuals older than 15 years who are illiterate >11.1%** | 1.04 (1.00-1.08) | 1.34 (1.03-1.70) | 1.34 (1.03-1.70) | 1.07 (0.89-1.28) | 1.01(0.98-1.03) |
| **Total fertility rate>2.32** | 1.07 (1.03-1.10) | 1.11 (0.95-1.30) | 1.11 (0.95-1.30) | 1.00 (0.87-1.15) | 1.06(1.04-1.08) |

**Models Equation**

Panel data post-sample forecasting is a type of forecasting which uses the time-series dimension of the dataset and forecasts the values of variables, both dependent and independent, from existing observations.^6,7^ Fixed effects negative-binomial longitudinal (panel) regression models and their parameters obtained from ex-post impact evaluations were used to perform a post-sample forecasting of U5MRs and U5HRs.^1,2^

Each outcome, for a specific year and specific municipality, was estimated as the product of the FE term of the municipality, the independent variables with their effects expressed as rate ratios (RR), and the dummy time variable with its RR, according to the following equation:

*Log* (*U5MR_it_)= α_i_ + β_1_ BFP _it_+ β_2_ ESF _it_+ β_k_ X_k it_ + β_3_t*

Where

*t* refers to the year and *i* refers to an individual municipality,

*U5MR_it_* is the U5MR in municipality *i* in year *t*,

*BFP _it_* is the coverage of BFP in municipality *i* in year *t* with a coefficient of *β_1_,*

*ESF _it_* is the coverage of ESF in municipality *i* in year *t* with a coefficient of *β_2_,*

*X_kit_* refers to each covariate (*k)* for municipality *i* in year *t* with coefficient *β_k_,*

*t* is the time dummy variable with coefficient *β_3_,*

*α_i_* is the fixed effect (time-invariant) term for each municipality.

Time was included in the model to capture omitted variables which could contribute to trends in U5MRs and U5HRs affecting all municipalities.

**Calibration of the models**

As explained above, all parameters of the models were derived from a pre-existing retrospective dataset and ex-post impact evaluation.^1^ The only calibrated variable of the model was the effect size of the time variable, representing U5MRs secular trends not captured by the adjusting variables of the regression, and which are assumed to change between different decades and economic cycles. The calibration was performed through comparison, for the period 2010-2014 (before the beginning of the economic crisis), of the model forecast U5MRs with actual U5MRs obtained from the Mortality Information System (*Sistema de Informação sobre Mortalidade (*SIM)), in a subset of 1,646 municipalities with adequate vital statistics reporting used in previous studies.^1,2^ These municipalities do not suffer from death undernotification and are usually less poor than the rest of the country. A descriptive analysis of the retrospective dataset in 2000 showed that poverty rate was 26.1% in the 1,646 municipalities with adequate vital information and 47.5% in the rest of the municipalities. As shown in some studies more developed and less poor municipalities have lower U5MR decreasing trends than the rest of the municipalities.^1,8,9^ To address this we calibrated the model using mean U5MR in the quintile of municipalities with lower poverty rates and introduced a trend correction factor. The trend correction factor has been calculated as the ratio between the linear time trend of the mean U5MR of all municipalities and the linear time trend of the U5MR of the municipalities quintile with lower poverty rate, obtained by the subgroup analysis module used for studying the inequalities between counties. The model with the lower Sum of Squared Errors between its U5MR linear trend and the U5MR linear trend of the 1,646 municipalities with adequate vital information during the period 2010-2014 had a coefficient for the regression time variable of 0.018, as shown in Table B.

**Table B. Sum of Squared Errors (SSE) between the linear trend coefficients of the mean U5MR in the municipalities with adequate vital information and the simulated mean U5MR of the quintile of municipalities with lower poverty rates for the period 2010-2014.**

| **Time Variable Coefficient of the Model** | **Trend of the Mean U5MR of Municipalities Adequate Information** | **Trend of the Simulated Mean U5MR of** **Municipality Quintile with Lower Poverty Rates** | **SSE** | **Trend of the** **Simulated Mean U5MR of All Municipalities** | **Simulated Mean U5MR of All Municipalities vs Municipality Quintile with Lower Poverty Rates** |
| --- | --- | --- | --- | --- | --- |
| 0.015 | -0.320 | -0.282 | 1.44E-03 | -0.728 | 2.58 |
| 0.016 | -0.320 | -0.295 | 1.02E-01 | -0.746 | 2.52 |
| 0.017 | -0.320 | -0.310 | 1.00E-04 | -0.766 | 2.47 |
| 0.018 | -0.320 | -0.325 | 2.50E-05 | -0.784 | 2.41 |
| 0.019 | -0.320 | -0.337 | 2.89E-04 | -0.804 | 2.39 |
| 0.020 | -0.320 | -0.353 | 1.09E-03 | -0.822 | 2.33 |

For the analysis of the three causes of under-five mortality (and for the under-five hospitalization rates) the time variable coefficient was obtained multiplying 0.018 with the ratio between the time trend of U5MR from the specific disease (or for hospitalization) in the period 2010-2014 and the time trend of U5MR for all causes for the same period, both obtained from data of the Mortality Information System (*Sistema de Informação sobre Mortalidade (*SIM). This calculation was based on the consideration that all outcomes were under the same expositions - with similar effect sizes – in the period 2010-2014.

**Internal Validation of the model**

Internal validity of the model was assessed fitting the fixed effects negative binomial multivariate regression described above - and used for the microsimulation - on the synthetic dataset created for the period 2010-2030, and verifying that the obtained coefficients for each variable were the same than the ones introduced as inputs in the model (and derived from the retrospective impact evaluation).

**External Validation of the Models**

The external validation of the model was undertaken comparing the overall national U5MR forecast by the microsimulation with the official Brazilian U5MR estimates during the years 2010-2013,^5^ which are the most up-to-date available, and estimating the linear regression and the correlation coefficients (R^2^) of predicted vs observed values, as shown in Figure C.

Moreover it was verified that all points of official U5MR estimates where included in the 95% Credible Intervals of our simulation.

National geographical patterns, obtained projecting the municipality variables on the country map, and correlation structures of all the variables of the model at the beginning and at the end of the simulation, have been used as robustness analysis to verify if important and non-expected variations were introduced by the simulation. Brazil is characterized by specific areas of poverty and socioeconomic vulnerability, which correspond closely to several health outcome patterns, in particular child mortality. Figure D shows that geospatial patterns will remain considerably similar even with changes in the values of the variables between 2010 and 2030.

**Fig C. Linear regression and correlation coefficient (R^2^) of predicted vs observed values, and trend of the simulated overall U5MR with its CIs vs the official Brazilian U5MR estimates for the period 2010-2013.**


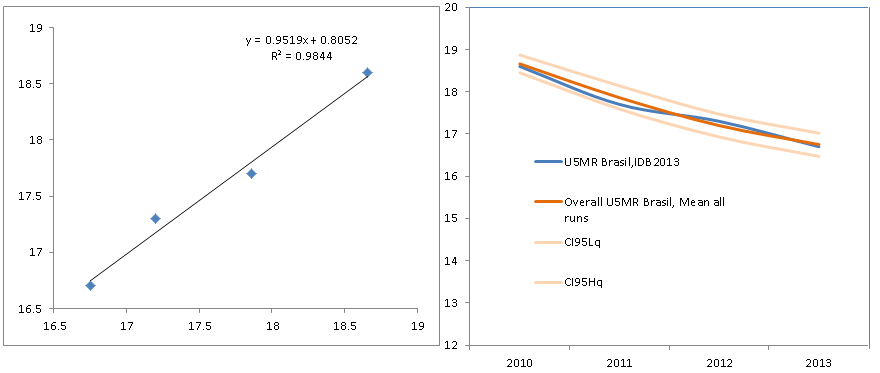


**Fig D. Geospatial patterns of U5MRs and Poverty rates in scenario of medium economic crisis and maintenance of the social protection.**


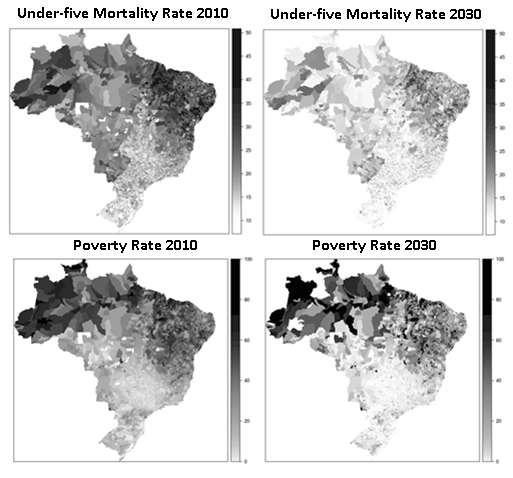


**Summary of Results**

Modelled scenarios: economic crisis and policy response

Tables and Figures illustrating the results are available in the main manuscript.

Under all economic crisis scenarios, poverty rates are forecast to increase and income per capita to fall in the coming years. In economic scenario 1 (a milder and shorter crisis), decreases in income and increases in poverty are estimated to cease from 2018 onwards, whilst for scenario 2 (a stronger crisis) this occurs two years later in 2020, and in scenario 3 in 2022. Other socio-economic variables included in the analysis follow historical trends over time showing reductions in illiteracy, declining fertility, and improvements in access to sanitation.

In all economic crisis and policy response scenarios, mean U5MRs are forecast to continue declining, although the magnitude of these reductions varies. In 2015 at the beginning of the economic crises, we observe a sharp slowing in annual U5MR reduction (0.2% a year) in comparison with the 2010-2015 period (0.8% a year) due to increasing poverty rates and falling incomes.

From 2017 onwards the two policy response scenarios are associated with different U5MRs. Continuing austerity and the concomitant reductions ESF and BFP coverage will slow the decline in U5MR relative to maintaining social protection. In economic crisis scenario 1 (a milder crisis), which consequently requires smaller increases in BFP and ESF coverage under the scenario of maintenance of social protection, the U5MR by 2030 would be 6.98% (CI:5.26-9.67%) lower compared to the austerity scenario with 13,954 (CI: 4,559-23,418) averted under-five deaths along the period. In the scenario 2 of deeper crisis with maintenance of social protection, the U5MR will be 8.57% (CI: 6.88%-10.24%) lower in 2030 than under austerity, and cumulative differences in mortality rates between 2017-2030 correspond to 19,732 (CI:10,207-29,285) averted under-five deaths under the social protection scenario. In economic crisis scenario 3 (a stronger and longer crisis) the U5MR by 2030 would be 9.53% (CI:7.92-11.17%) lower compared to the austerity scenario with 23,424 (CI:13,829-32,880) averted under-five deaths during the study period.

Attenuations in annual reductions in U5MR and U5HR are observed for both policy responses scenarios, but under the policy response scenario of austerity increases in the U5MR are actually forecast for diarrhoeal diseases and malnutrition. Under policy response scenario of maintained social protection mortality for these causes would continue to decline, albeit at a lower rate, resulting in U5MRs in 2030 that are 39.3% (CI:36.9%-41.8) and 35.8% (CI:31.5%-39.9%) lower respectively for diarrhoeal diseases and malnutrition than under austerity. For lower respiratory tract infections by 2030 the U5MR would be 8.5% (CI:4.1%-12.0%) lower under maintenance of social protection. Declines in U5HRs continue under both policy response scenarios, albeit at a slower rate under the austerity scenario. Under policy response scenario 2 (maintenance of social protection) there would be greater reductions in the U5HR which by 2030 would be 3.1% (CI 0.9%-5.1%) lower, corresponding to 123,549 (CI 21,248-226,292) averted U5 hospitalizations along the study period 2017-2030.

Stratification and Inequality analysis

Stratification of U5MR trends by quintiles of municipal level poverty shows that the effects of the maintenance of social protection are greatest in the poorest municipalities, reaching a mortality reduction of 11.01% (CI: 7.97%-13.83%) in 2030 in the fifth quintile versus a non-significant 4.10% (CI:-0.69%-8.61%) reduction in the richest quintiles. Concerning inequalities, while the Concentration Index of U5MR among municipalities will not decrease in the case of austerity (0.129 (CI: 0.122-0.137) in 2015 vs 0.128 (CI: 0.121-0.135) in 2030), under the maintenance of social protection it will be 0.111 (CI: 0.104-0.119) in 2030, 13.3% (CI: 5.6% -21.8%) lower than under austerity.

Sensitivity Analyses

a. Varying economic crisis intensity

A recent microsimulation study from the WB ^10^ simulated the increase in poverty rates for the years 2016 and 2017 of 9.8% and 10.3% for the worst scenario and 9.7% and 9.8% for the milder crisis scenario and indicated a need for increases in the Federal Budget in 2017 for poverty-relief programmes, in particular the Bolsa Familia Programme.

To evaluate how a lengthening of the economic crisis could affect mortality rates we additionally modelled the impact of austerity and social protection maintenance where the crisis lasts for 9, 11 and 13 years (Table C).

**Table C. U5MRs Rate Ratios between Austerity Measures and Social Protection maintenance according to different length of the economic crisis.**

|  | **9 years** | | **11 years** | | **13 years** | |
| --- | --- | --- | --- | --- | --- | --- |
| **Years** | **RR** | **Credible**  **Intervals** | **RR** | **Credible**  **Intervals** | **RR** | **Credible**  **Intervals** |
|  |  |  |  |  |  |  |
| 2015 | 1 | (0.9819-1.0183) | 1.0007 | (0.9826-1.0190) | 1 | (0.9823-1.0184) |
| 2016 | 0.9997 | (0.9812-1.0184) | 1.0008 | (0.9825-1.0197) | 0.9999 | (0.9820-1.0176) |
| 2017 | 0.9866 | (0.9681-1.0046) | 0.9867 | (0.9694-1.0043) | 0.9862 | (0.9686-1.0040) |
| 2018 | 0.9747 | (0.9568-0.9931) | 0.9753 | (0.9573-0.9927) | 0.9743 | (0.9568-0.9912) |
| 2019 | 0.9634 | (0.9457-0.9814) | 0.9641 | (0.9469-0.9809) | 0.9632 | (0.9473-0.9805) |
| 2020 | 0.9500 | (0.9328-0.9672) | 0.9505 | (0.9342-0.9661) | 0.9500 | (0.9326-0.9668) |
| 2021 | 0.9395 | (0.9220-0.9565) | 0.9400 | (0.9235-0.9572) | 0.9392 | (0.9228-0.9570) |
| 2022 | 0.9300 | (0.9130-0.9485) | 0.9306 | (0.9129-0.9478) | 0.9295 | (0.9137-0.9463) |
| 2023 | 0.9211 | (0.9049-0.9391) | 0.9215 | (0.9049-0.9386) | 0.9210 | (0.9046-0.9370) |
| 2024 | 0.9177 | (0.9017-0.9351) | 0.9129 | (0.8963-0.9301) | 0.9117 | (0.8954-0.9286) |
| 2025 | 0.9124 | (0.8958-0.9293) | 0.9038 | (0.8877-0.9214) | 0.9030 | (0.8870-0.9194) |
| 2026 | 0.9083 | (0.8918-0.9254) | 0.9008 | (0.8849-0.9172) | 0.8951 | (0.8796-0.9114) |
| 2027 | 0.9046 | (0.8869-0.9213) | 0.8980 | (0.8824-0.9143) | 0.8887 | (0.8729-0.9059) |
| 2028 | 0.9020 | (0.8854-0.9186) | 0.8962 | (0.8805-0.9129) | 0.8887 | (0.8723-0.9050) |
| 2029 | 0.9003 | (0.8839-0.9166) | 0.8950 | (0.8787-0.9102) | 0.8880 | (0.8724-0.9047) |
| 2030 | 0.8985 | (0.8828-0.9162) | 0.8940 | (0.8777-0.9111) | 0.8872 | (0.8708-0.9040) |

Differential poverty rate increases were also tested according to municipal characteristics at baseline (2010) with larger increases in poverty in poorer municipalities and smaller increases in the wealthier municipalities. No relevant differences in terms of RR where observed in comparison with the findings presented in the main text.

b. Varying policy response intensities

As sensitivity analysis, we modelled additional austerity scenarios where percentage reductions in coverage of BFP and ESF were varied (Table D). Under all these scenarios austerity measures were associated with important excess in avoidable child mortality.

**Table D. U5MRs Rate Ratios between Austerity Measures and SP Strengthening for scenario 2 according to the percent yearly decrease of BFP and ESF due to austerity measures.**

|  | **BFP & ESF 3% reduction/y** | | **BFP & ESF 2% reduction/y** | | **BFP & ESF 1% reduction/y** | | **BFP 2% & ESF 4% reduction/y** | | **BFP 4% & ESF 2% reduction/y** | |
| --- | --- | --- | --- | --- | --- | --- | --- | --- | --- | --- |
| **Years** | **RR** | **Credible Intervals** | **RR** | **Credible Intervals** | **RR** | **Credible Intervals** | **RR** | **Credible Intervals** | **RR** | **Credible Intervals** |
|  |  |  |  |  |  |  |  |  |  |  |
| 2015 | 1.0002 | (0.9831-1.0192) | 1.0005 | (0.9825-1.019) | 1.0002 | (0.9831-1.0192) | 1.0005 | (0.9825-1.019) | 1.0005 | (0.9825-1.019) |
| 2016 | 1.0005 | (0.9831-1.0197) | 1.0001 | (0.9829-1.0174) | 1.0005 | (0.9831-1.0197) | 1.0001 | (0.9829-1.0174) | 1.0001 | (0.9829-1.0174) |
| 2017 | 0.988 | (0.9713-1.0069) | 0.9895 | (0.9718-1.0078) | 0.9903 | (0.9737-1.0091) | 0.9889 | (0.9712-1.0072) | 0.9876 | (0.9699-1.006) |
| 2018 | 0.978 | (0.9596-0.996) | 0.9803 | (0.9624-0.9975) | 0.9832 | (0.9647-1.0013) | 0.979 | (0.9612-0.996) | 0.9761 | (0.9584-0.9929) |
| 2019 | 0.9689 | (0.952-0.9864) | 0.9733 | (0.9553-0.9906) | 0.9772 | (0.9599-0.9947) | 0.9709 | (0.9527-0.9881) | 0.9667 | (0.949-0.9841) |
| 2020 | 0.9626 | (0.9462-0.9793) | 0.9684 | (0.9514-0.9862) | 0.9741 | (0.9576-0.9911) | 0.9643 | (0.9474-0.9818) | 0.96 | (0.9434-0.9777) |
| 2021 | 0.9592 | (0.9421-0.9772) | 0.9671 | (0.95-0.9844) | 0.9745 | (0.957-0.9928) | 0.9604 | (0.9437-0.9775) | 0.9573 | (0.9402-0.9746) |
| 2022 | 0.9571 | (0.9399-0.9738) | 0.9675 | (0.9506-0.9856) | 0.9761 | (0.9583-0.9928) | 0.9573 | (0.9405-0.9754) | 0.9571 | (0.9405-0.9751) |
| 2023 | 0.9548 | (0.9375-0.9716) | 0.9662 | (0.9497-0.9835) | 0.9774 | (0.9599-0.9947) | 0.9516 | (0.9357-0.9689) | 0.9558 | (0.9395-0.9728) |
| 2024 | 0.952 | (0.9347-0.9693) | 0.9656 | (0.9484-0.9824) | 0.9784 | (0.9607-0.9963) | 0.9461 | (0.9293-0.9624) | 0.9552 | (0.9382-0.9719) |
| 2025 | 0.9487 | (0.9314-0.966) | 0.9645 | (0.9464-0.9816) | 0.9791 | (0.9614-0.9974) | 0.9403 | (0.9227-0.9569) | 0.9539 | (0.9356-0.9708) |
| 2026 | 0.9454 | (0.9287-0.9626) | 0.9635 | (0.9467-0.9806) | 0.9802 | (0.9629-0.9981) | 0.9356 | (0.9194-0.9521) | 0.9524 | (0.9357-0.9695) |
| 2027 | 0.9409 | (0.9245-0.9581) | 0.9627 | (0.945-0.9806) | 0.9802 | (0.9635-0.9983) | 0.9327 | (0.9156-0.9506) | 0.9505 | (0.9329-0.9686) |
| 2028 | 0.937 | (0.921-0.9528) | 0.9616 | (0.9436-0.98) | 0.9808 | (0.9642-0.9976) | 0.9312 | (0.9138-0.9488) | 0.9481 | (0.9304-0.9665) |
| 2029 | 0.9336 | (0.9174-0.952) | 0.9599 | (0.9429-0.9778) | 0.9817 | (0.9644-1.0012) | 0.9306 | (0.9141-0.9478) | 0.9449 | (0.928-0.9623) |
| 2030 | 0.9303 | (0.9142-0.9465) | 0.9586 | (0.9419-0.9758) | 0.9819 | (0.9648-0.9988) | 0.9313 | (0.9148-0.9478) | 0.9418 | (0.9254-0.9586) |
|  |  |  |  |  |  |  |  |  |  |  |

c. Varying U5MRs secular trends

In order to verify that an inaccurate calibration of the time coefficient or that unexpected secular trends would not change the findings of the study, models have been run with the same inputs and parameters but different time trend coefficients – in terms of yearly percent U5MR reduction- and results in terms of effects of austerity measures were identical, as shown in Table E.

**Table E. Effects of austerity measures in terms of rate ratios (RR) for the medium length economic crisis scenario and different U5MR secular trends at the year 2030.**

|  | **Scenario 1 of Economic Crisis, Austerity vs Social Protection** | |
| --- | --- | --- |
| **Coefficient of Time Variable** | **RR** | **Credible Intervals** |
| 0.015 | 0.9142 | (0.8983-0.9302) |
| 0.020 | 0.9143 | (0.8982-0.9301) |
| 0.025 | 0.9142 | (0.8983-0.9303) |

**Fig E. U5MR projections for the medium length economic crisis scenario, the two different policy options and different U5MR secular trends.**


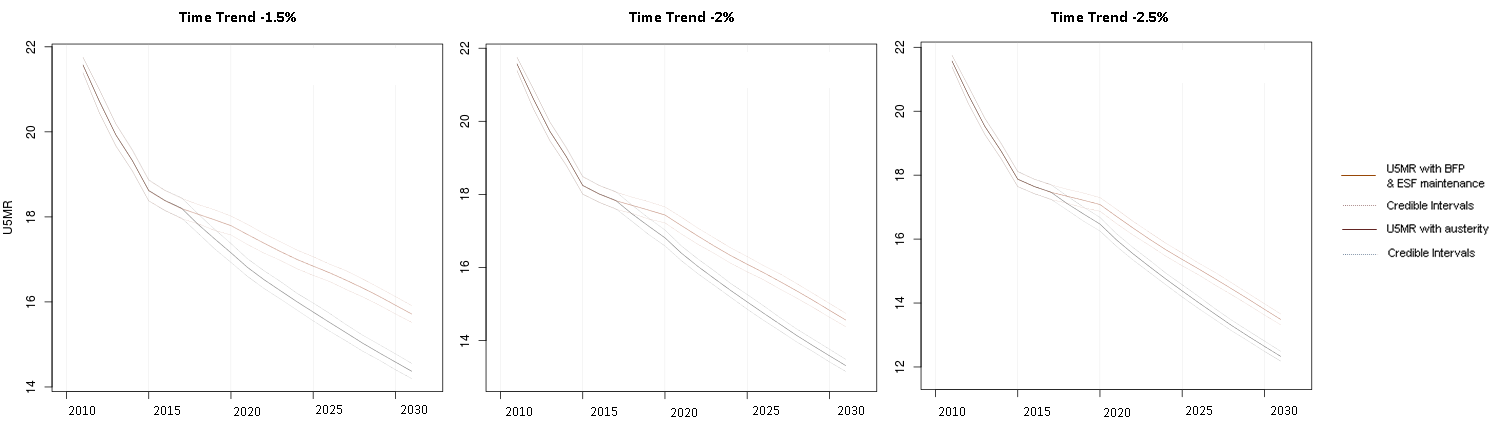


The Rate Ratio was robust to changes in secular trends values and was verified for the specific causes of under-five mortality and for under-five hospitalization rates, and for different secular trends modifications in the economic crisis period.

d. Comparing Austerity and a shorter economic crisis versus maintenance of Social Protection and a longer crisis

We have simulated - with our models and our parameters - the possibility that fiscal austerity measures may shorten the economic crisis in Brazil, while the maintenance of social protection would extend this.

The scenario of fiscal austerity and mild economic crisis described above was compared with a scenario of maintenance of social protection and a longer economic crisis.

As shown in Figure F, with an increase of poverty and decrease of income until 2030 – following the exponential decay and parameters used for the previous simulated scenarios - the maintenance of social protection will still produce a 4.43% (CI: 2.79%-6.32%) reduced U5MR in 2030 in comparison with fiscal austerity, even if the latter would be associated with a milder and shorter economic crisis (up to 2018).

**Fig F. U5MR projections for the medium length economic crisis scenario, the two different policy options and different U5MR secular trends.**


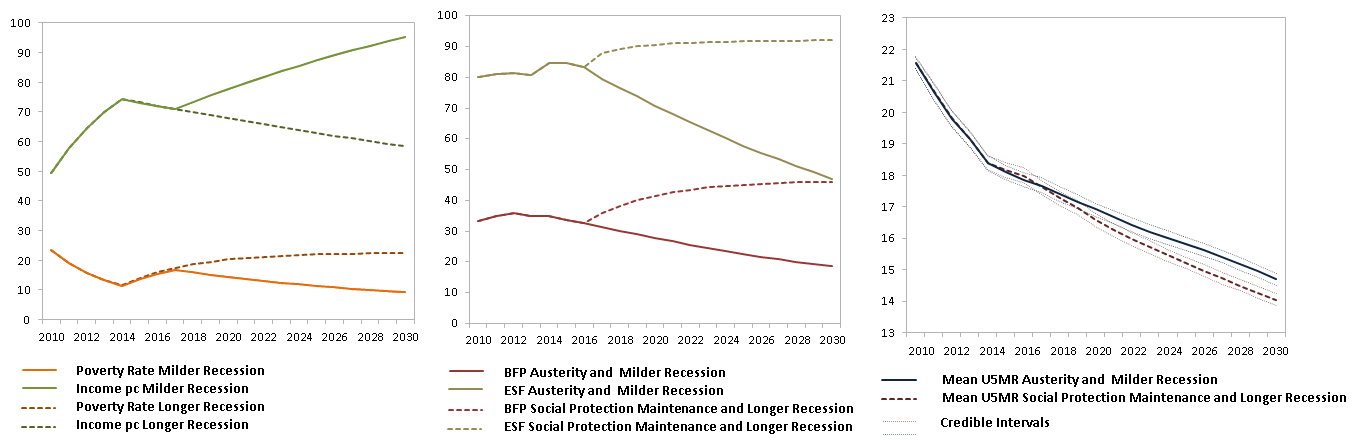


**Main limitations**

The main limitation of the study is the uncertainty around the future macroeconomic scenarios in Brazil, due to the current extremely unstable political and economic situation, which creates uncertainty around the forecasting of poverty rates, income, and the other independent variables. For that reason several scenarios have been simulated in sensitivity analyses which produced comparative findings. Another limitation is that the modelling of austerity measures is focused on BFP and ESF as there is strong evidence that these policies confer protective effects for childhood morbidity and mortality from previous studies.^1,2^ Our estimates of the impact of austerity measures on child health are probably conservative as they do not reflect constraints in other areas of public spending e.g. education, housing and other welfare programmes which have known impacts on poverty and health. Moreover, austerity measures recently enshrined in the constitution of Brazil means that public spending will only increase in line with inflation, which will not account for the demographic growth of the population, its ageing processes, and growing costs associated with new healthcare treatments and technologies.^11,12,13^ Another limitation of the study is that we do not model the impact of the increased coverage of BFP on poverty rate dynamics, assuming that poverty rates influence BFP coverage and not the contrary. This is mainly due to the assumption that WB simulations of poverty increase during economic crisis already account for BFP effects, and because reliable parameters were not available at the moment of writing.

**Sources of funding and their role**

The development of the model was funded by the Wellcome Trust Training Fellowships in Public Health and Tropical Medicine scheme (Grant reference number: 109949/Z/15/Z). CM is funded by a Research Professorship award from the National Institute for Health Research. The funding was not specific for the study described in this article. The funder had no role in study design, data collection, data analysis, data interpretation, writing of the report, or in the decision to submit this article for publication. All researchers´ decisions have been entirely independent from funders.

**S3 Text References**

1. Rasella D, Aquino R, Santos CAT, Paes-Sousa R, Barreto ML. Effect of a conditional cash transfer programme on childhood mortality: a nationwide analysis of Brazilian municipalities. The Lancet 2013; 382(9886): 57-64.
2. Rasella D, Aquino R, Barreto ML. Reducing childhood mortality from diarrhea and lower respiratory tract infections in Brazil. Pediatrics 2010; 126(3): e534-e40.
3. IBGE. Instituto Brasileiro de Geografia e Estatística (IBGE). Censos Demográficos. 2017. http://www.ibge.gov.br/ (accessed 08/04 2017).
4. Ministerio do Desenvolvimento Social e Combate a Fome. Matriz de Informação Social. 2017. http://aplicacoes.mds.gov.br/sagi/mi2007/tabelas/mi_social.php (accessed 08/04 2017).
5. Ministério da Saúde Brasil. DATASUS. 2017. http://tabnet.datasus.gov.br/ (accessed 08/04 2017).
6. Fiebig D, Johar M, Forecasting with Micro Panels: The Case of Health Care Costs. Journal of Forecasting, 2016, 36:1-15
7. Baltagi H, Forecasting with panel data. Journal of Forecasting, 2008, 27:153-161.
8. Barreto ML, Rasella D, Machado DB, Aquino R, Lima D, Garcia LP, Boing AC, Santos J, Escalante J, Aquino EM, Travassos C. Monitoring and evaluating progress towards Universal Health Coverage in Brazil. PLoS Med. 2014;11(9):e1001692.
9. Rasella D, Harhay MO, Pamponet ML, Aquino R, Barreto ML. Impact of primary health care on mortality from heart and cerebrovascular diseases in Brazil: a nationwide analysis of longitudinal data. BMJ 2014; 349: g4014.
10. Skoufias E, Nakamura S, Gukovas RM. Safeguarding against a reversal in social gains during the economic crisis in Brazil. Working Paper 112896. Washington, DC, USA: The World Bank, 2017.
11. Paiva AB, Mesquita ACS, Jaccoud L, Passos L. [The new tax regime and its implications for social assistance policy in Brazil.] [Portuguese]. Technical Note No.27. Brasilia, Brazil: Instituto de Pesquisa Econômica Aplicada (IPEA), 2016.
12. Vieira FS, Benevides RPdSe. [The impacts of the New Tax Regime for the financing of the Unified Health System and for the realization of the right to health in Brazill] [Portuguese]. Technical Note No.28. Brasilia, Brazil: Instituto de Pesquisa Econômica Aplicada (IPEA), 2016.
13. Rossi P, Dweck E. Impacts of the new fiscal regime on health and education. Cadernos de saude publica 2016; 32:12-15.
