# Supplementary material for: Child morbidity and mortality associated with alternative policy responses to the economic crisis in Brazil: A nationwide microsimulation study
Source: PLoS Med. 2018 May 22;15(5):e1002570. doi: 10.1371/journal.pmed.1002570 (PMC5963760; doi:10.1371/journal.pmed.1002570)
Supplement: S4 Text — (DOCX) [file pmed.1002570.s004.docx]

**S4 Text. The three economic crisis scenarios: comparative results**

In order to compare the findings of the economic crisis scenario 2 (medium crisis) with the other two scenarios presented in the text, a milder and shorter (3 years) economic crisis and one with same intensity of scenario 2 but longer (7 years), below are presented the findings for causes of under-five mortality and by quintiles of poverty as done for scenario 2 in the manuscript (Fig 5, Fig 6 and Table 3 of the manuscript). As detailed in the main text, the intensity of the crisis in terms of yearly percent increase of poverty rate (milder for scenario 1 and stronger for scenario 2 and 3) is based on Brazil-specific World Bank microsimulation of poverty increases due to the actual crisis [1], the lengths – in terms of years of poverty with an increasing trend, not GDP - have been chosen in order to forecast a more optimistic, a medium and a more pessimistic scenario. These lengths appear as the most reasonable at the moment of writing, considering the recent unemployment and poverty rate measurements, the high income inequality of the country and the short and medium-term political instability [2-9].

**Table A. Under-five rate ratios between the two policy response options by the three economic crises scenarios in 2030.**

|  | | **Scenario 1** | | **Scenario 2** | | **Scenario 3** | |
| --- | --- | --- | --- | --- | --- | --- | --- |
| ***Rates*** | | **RR** | **Credible**  **Intervals** | **RR** | **Credible**  **Intervals** | **RR** | **Credible**  **Intervals** |
|  | |  |  |  |  |  |  |
| Diarrhoeal Diseases | 0.6770 | | (0.6503-0.7041) | 0.6066 | (0.5824-0.6314) | 0.5703 | (0.5473-0.5941) |
| Malnutrition | 0.7324 | | (0.6838-0.7791) | 0.6415 | (0.6001-0.6861) | 0.5930 | (0.5567-0.6319) |
| Lower Respiratory Tract Infections | 0.9494 | | (0.9130-0.9911) | 0.9155 | (0.8789-0.9540) | 0.8966 | (0.8602-0.9314) |
| U5 Hospitalization | 0.9870 | | (0.9660-1.0082) | 0.9693 | (0.9488-0.9901) | 0.9592 | (0.9398-0.9804) |

**Table B. Rate Ratio between Concentration Indexes of Under-five Mortality Rate by municipal poverty rate between the two policy response options by the three economic crises scenarios in 2030.**

|  | **Scenario 1** | | | | **Scenario 2** | | | **Scenario 3** | | |
| --- | --- | --- | --- | --- | --- | --- | --- | --- | --- | --- |
|  | **RR** | | **Credible**  **Intervals** | | **RR** | **Credible**  **Intervals** | | **RR** | **Credible**  **Intervals** | |
|  |  | |  |  |  |  |  |  |  |  |
| Concentration Index | | 0.909 | (0.8292-0.9917) | | 0.8670 | (0.7923-0.9439) | | 0.8546 | (0.7816-0.9299) | |

**Fig A: Mean municipal under-five mortality rates for selected causes and under-five hospitalization rate for the period 2010-2030 under economic crisis all three economic crisis scenarios with the two policy responses (austerity and maintenance of the social protection).**

**
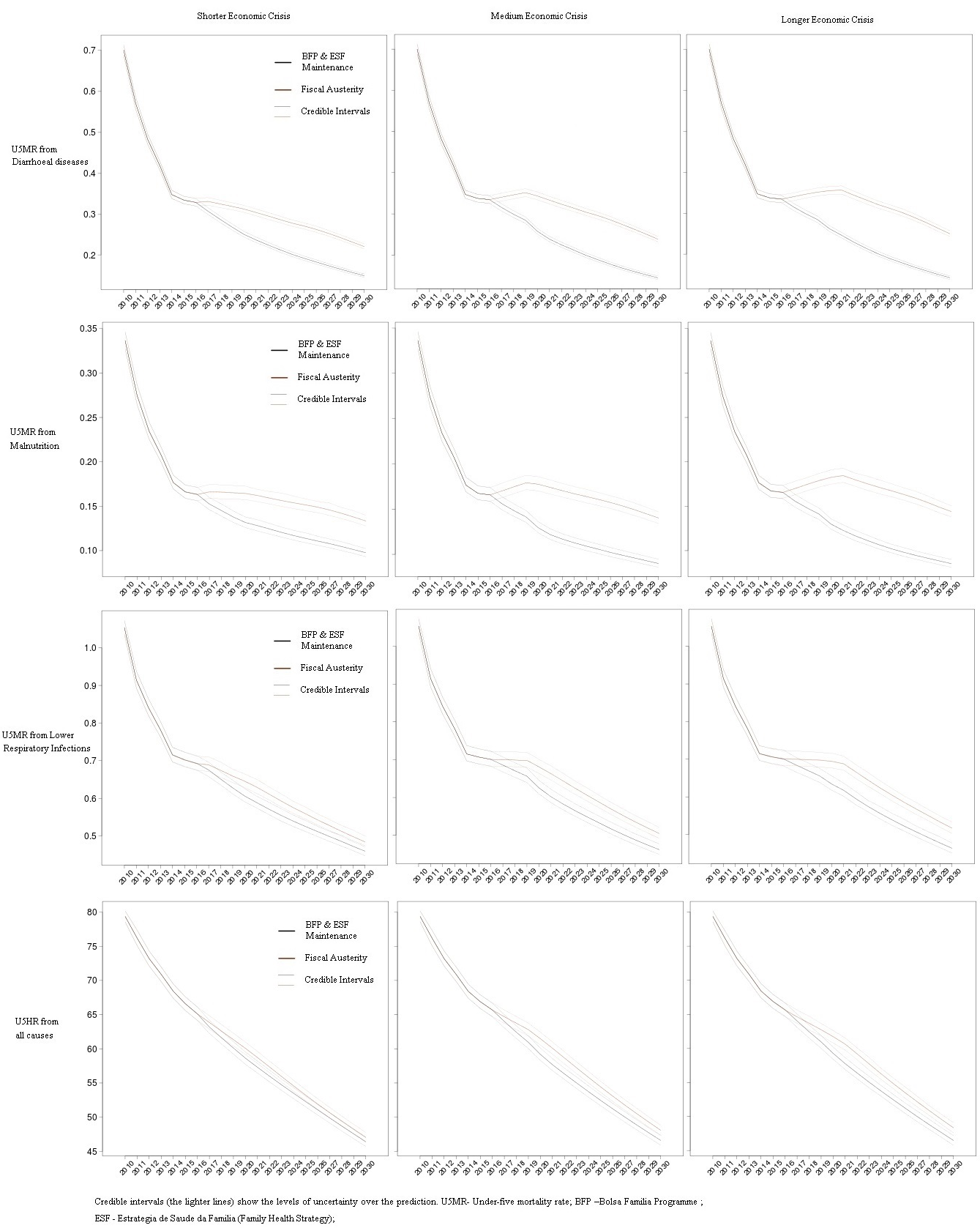
**

**Fig B: Mean municipal under-five mortality rates by poverty quintiles of municipalities for 2010-2030 under all three economic crisis scenarios and for both policy responses (austerity and social protection maintenance).**

**
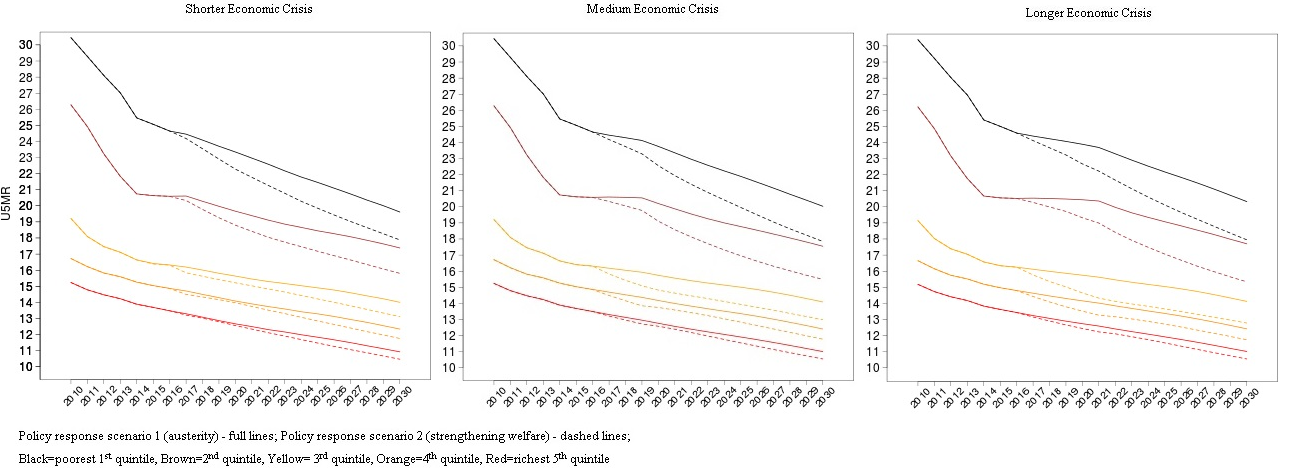
**

**S4 Text References**

1. Skoufias E, Nakamura S, Gukovas RM. Safeguarding against a reversal in social gains during the economic crisis in Brazil. Working Paper 112896. Washington, DC, USA: The World Bank, 2017.
2. International Monetary Fund. World Economic Outlook, October 2017 Seeking Sustainable Growth: Short-Term Recovery, Long-Term Challenges. Washington DC, USA: International Monetary Fund (IMF), 2017.
3. Fosu AK. Growth, inequality, and poverty reduction in developing countries: Recent global evidence. *Research in Economics* 2017; **71**(2): 306-336.
4. Morgan M. Extreme and Persistent Inequality: New Evidence for Brazil Combining National Accounts, Surveys and Fiscal Data, 2001-2015. WID.world Working Paper, 2017/12.

http://wid.world/wp-content/uploads/2017/09/Morgan2017BrazilDINA-.pdf (accessed 08/10 2017).

1. The Economist. Challenges ahead despite brighter short-term economic data. October 25th 2017. http://country.eiu.com/article.aspx?articleid=796011863&Country=Brazil&topic=Politics# (accessed 30/10 2017).
2. Financial Times. Brazil’s economy: from zombie to walking dead. Available at: https://www.ft.com/content/f3d2cd90-1e46-11e8-aaca-4574d7dabfb6
3. Uol. Economia. Taxa média de desemprego sobe em 2017 e é a maior desde 2012, diz IBGE. Available at: https://economia.uol.com.br/empregos-e-carreiras/noticias/redacao/2018/01/31/desemprego-quarto-trimestre-ibge.htm
4. G1. Globo. Prévia' do PIB do Banco Central registra queda de 0,56% em janeiro, Available at: https://g1.globo.com/economia/noticia/previa-do-pib-do-banco-central-inicia-2018-com-queda-de-056-em-janeiro.ghtml
5. Valor Economico. Pobreza extrema aumenta 11% e atinge 14,8 milhões de pessoas in 2017. Available at: http://www.valor.com.br/brasil/5446455/pobreza-extrema-aumenta-11-e-atinge-148-milhoes-de-pessoas
